# Large and Stable Nanopores Formed by Complement Component 9 for Characterizing Single Folded Proteins

**DOI:** 10.1021/acsnano.4c11666

**Published:** 2025-01-28

**Authors:** Wachara Chanakul, Anasua Mukhopadhyay, Saurabh Awasthi, Anna D. Protopopova, Alessandro Ianiro, Michael Mayer

**Affiliations:** † Adolphe Merkle Institute, University of Fribourg, Fribourg 1700, Switzerland; ‡ National Center for Competence in Research Bio-Inspired Materials, University of Fribourg, Fribourg 1700, Switzerland

**Keywords:** protein nanopore, large biological nanopore, single-molecule, membrane attack complex, complement component 9, amphipol, resistive pulse recording, change in protein conformation

## Abstract

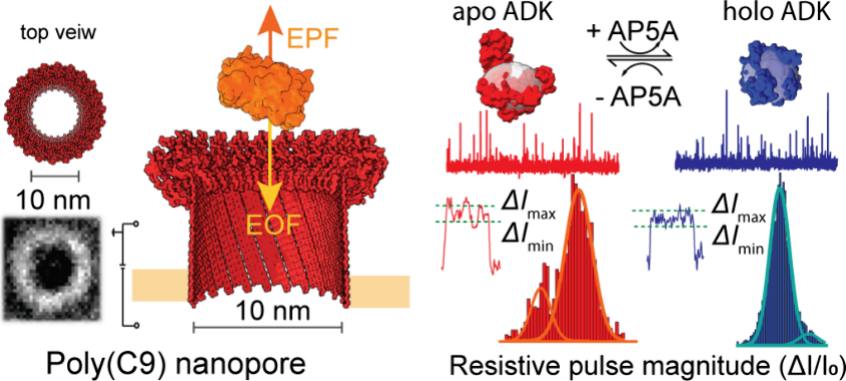

Biological nanopores offer a promising approach for single-molecule analysis of nucleic acids, peptides, and proteins. The work presented here introduces a biological nanopore formed by the self-assembly of complement component 9 (C9). This exceptionally large and cylindrical protein pore is composed of 20 ± 4 monomers of C9 resulting in a diameter of 10 ± 4 nm and an effective pore length of 13 nm. These poly(C9) pores remain stable for up to 30 min without indications of gating, flickering, or clogging across a range of transmembrane voltages (−150 to +150 mV) and ionic strengths (50 to 1000 mM). At physiologic pH, the ring-shaped distribution of negative and positive surface charges in the lumen of the pore enables capture of analyte proteins by electro-osmotic flow and leads to residence times of analyte proteins whose most probable values can exceed 300 μs. We used poly(C9) nanopores to determine the volume and shape of unlabeled folded proteins with molecular weights between 9 and 230 kDa with unprecedented accuracy in the context of resistive pulse recordings. Finally, poly(C9) pores made it possible to distinguish between the open and closed conformations of adenylate kinase based on differences in current modulations within resistive pulses and the corresponding differences in approximations of their shape. Thus, poly(C9) nanopores enable highly sensitive and accurate characterization of a wide range of natively folded proteins on a single molecule level.

## Introduction

Biological nanopores have become increasingly important analytical tools because they enable the characterization of unlabeled synthetic and biological molecules such as polymers,^1,2^ DNA,^3−5^ peptides,^6,7^ proteins,^8−14^ and small molecular analytes^15−17^ on a single molecule level. Biological nanopore sensors typically consist of two electrolyte-filled compartments (called cis- and trans-compartments) separated by a phospholipid or block copolymer membrane bearing a single proteinaceous pore. When a potential difference is applied across the membrane, ions in the electrolyte flow through the nanopore, resulting in a net ionic current. The entry of a (macro)molecule into the pore displaces highly conducting aqueous electrolyte from the pore lumen, leading to a transient decrease in the ionic current.^18^ This signal, known as a resistive pulse, can be analyzed to determine the sequence or structural properties of the analyte.^19,20^

Biological nanopores have been developed alongside with solid-state nanopores,^19,20^ which use an artificially manufactured pore in a synthetic membrane, which is often composed of silicon nitride, instead of a phospholipid membrane. Although solid-state nanopores can be prepared with a wide range of diameters and provide electrical measurements across a wide range of applied voltages and experimental conditions, their geometry and diameter along the length of the pore often vary from pore to pore and solid state nanopores tend to increase in diameter during experiments due to slow etching in the recording electrolyte. In contrast, biological nanopores surpass solid-state nanopores in several important aspects. These protein pores have a reproducible structure characterized at the atomic level, are typically resistant to clogging with the analyte, can be precisely modified using a range of molecular biology tools,^21^ and feature a small diameter that is hard to achieve reproducibly in solid-state nanopores.^22,23^

While small biological nanopores are required for DNA sequencing and are well established,^24,25^ larger ones for the structural characterization of natively folded proteins are still in early development.^13,26−28^ The two largest biological nanopores reported to date in the context of resistive pulse recordings are the two-component pleurotolysin toxin (PlyAB),^9^ which forms cylindrical pores with an internal diameter of 5.5 nm and a length of 10 nm, and the *Yersinia enterocolitica* pore-forming toxin (YaxAB),^27,28^ which forms conical pores with a 15 nm wide entrance and a 3.5 nm constriction. The PlyAB pore enables the detection of small, folded proteins up to approximately 65 kDa,^9^ while translocating larger proteins such as human serum albumin (66.5 kDa) and transferrin (76–81 kDa) requires further engineering of the nanopore lumen.^26^ The conical YaxAB pore exhibits a unique behavior in which the current blockade decreases with increasing size of the trapped protein.^27^ While the YaxAB pore has enabled the detection of relatively large proteins, such as a 125 kDa C-reactive protein,^27^ its application for accurately estimating the volume and shape of folded proteins remains limited.

**Figure 1:**
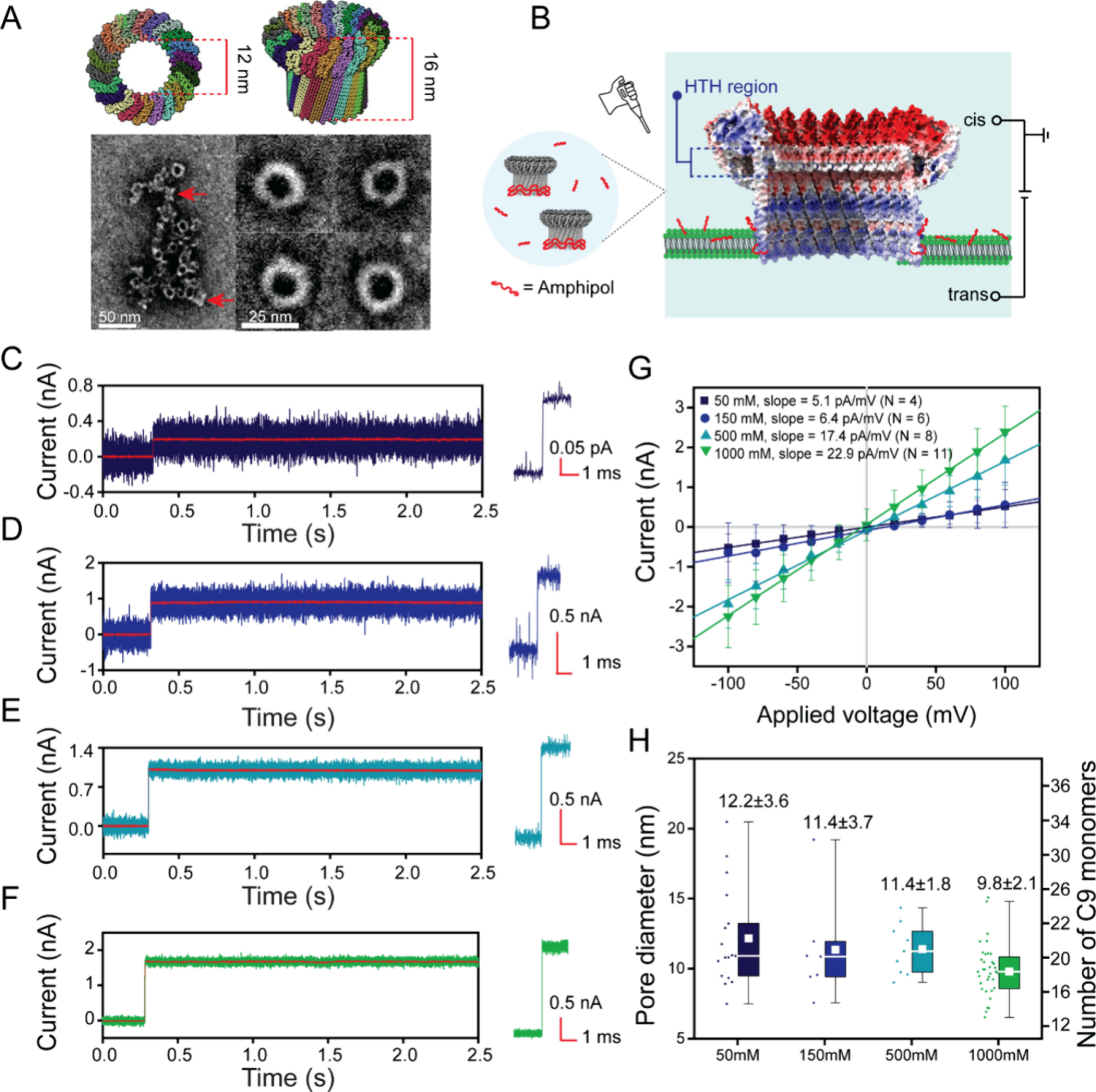
Characterization of the poly(C9) nanopore assembly and membrane reconstitution. (A) Overview of the structure of poly(C9). Top: cryo-EM structure with 22 identical subunits shown in different colors, top and oblique views (PDB 5FMW).^31^ Bottom: TEM images of poly(C9)-amphipol complexes showing characteristic top and side views. Side views are marked with arrows (B) Schematics of the pore insertion procedure. Poly(C9)-amphipol complexes are added to the cis compartment for spontaneous insertion of a poly(C9) pore. Nanopore lumen is colored by electrostatic potential with the red color corresponding to negative charge and the blue color to positive. Helix-turn-helix (HTH) region is labeled with dark blue lines. Lipid bilayer placement was guided by the cryo-EM structure (PDB 6DLW), where amphipols in the electron density map (EMD-7773) form a ring around the protein, matching the region otherwise shielded by the bilayer. (C–F) Single-pore insertions of poly(C9) into lipid bilayers under +100 mV applied potential with a recording buffer containing 10 mM HEPES pH 7.5 and 50 mM NaCl (C), 150 mM NaCl (D), 500 mM NaCl (E), or 1 M NaCl with 0.2 μM amphipol (F). All current recordings were collected with a sampling rate of 200 kHz and filtered with a Gaussian low-pass filter with a cutoff frequency of 100 kHz; recordings in red color show the same data after low-pass filtering at 10 kHz; both filter cutoff frequencies were chosen for display purpose in this figure, for details on cutoff frequencies used for quantitative data analysis, see Methods section. Moments of pore insertion are displayed on the right with an expanded time axis and a 50 kHz Gaussian low-pass filter. (G) Current–voltage (*I*–*V*) curves of the poly(C9) pore at different NaCl concentrations. Error bars represent the standard deviations calculated from a minimum of three repeats. (H) Estimations of the inner diameter of the poly(C9) pore at different NaCl concentrations assuming a published pore length of 16 nm.^30,43^ Mean values are shown by the solid white squares, the median values by horizontal lines. Box range corresponds to the 25th to 75th percentile, whereas the whisker range corresponds to the fifth to 95th percentile.

The work presented here demonstrates the use of complement component 9 (C9, MW = 70 kDa),^29−31^ a protein that is a part of the innate mammalian immune response, as a biological nanopore sensor for resolving the volume and shape of unlabeled folded proteins. *In vivo*, C9, along with four other complement proteins (C5, C6, C7, and C8) of the immune system, forms a membrane attack complex (MAC) on the surface of pathogenic cells.^32,33^ The MAC creates a pore in the plasma membrane of the targeted cell that allows molecules to diffuse freely across the membrane of the pathogen, leading to its death.^34,35^*In vitro*, C9 can self-assemble into tubular complexes with amphiphilic properties.^30,31,36−39^ These self-assembled C9 oligomers, termed poly(C9), have been elucidated *via* transmission electron microscopy (TEM)^36−39^ and cryogenic electron microscopy (cryo-EM),^30,31^ revealing a ring-like structure with a symmetrical arrangement of 22 ± 1 monomers of C9, an internal cross-sectional diameter of 12 nm, and a length of 16 nm (Figure 1A). Due to these unique characteristics, C9 is an attractive candidate of a biological nanopore for resistive pulse-based characterization of natively folded single macromolecules.

To facilitate the self-assembly of C9 to poly(C9) in aqueous solution and to enable its spontaneous single-step insertion into planar lipid bilayer, we utilized the polymeric surfactant amphipol A8–35.^30,31^ We characterized the poly(C9) nanopore, focusing on its dimensions, electrical stability, and noise properties. To evaluate its performance in resistive pulse recordings, we tested seven unlabeled, folded proteins with molecular weights ranging from 8.6 kDa to 97.2 kDa. This analysis provided precise estimates of protein volume and length-to-diameter ratios *m* (defined as *m* = A/B for an ellipsoid of revolution with axes A, B, B). Notably, we identified a high molecular weight subpopulation in one protein, likely corresponding to a dimer with a molecular weight close to 200 kDa. Furthermore, we distinguished the open and closed conformation of another protein by differences in approximations of their shape. These findings establish poly(C9) nanopores as a reliable tool for characterizing the volume and shape of single folded proteins, surpassing the accuracy of previous nanopore-based resistive pulse recording techniques.

## Results

### Self-Assembly and Membrane Reconstitution of Poly(C9)

To develop a C9-based nanopore for resistive pulse recordings, we began by assembling and characterizing poly(C9)-amphipol complexes in solution. Next, we created planar lipid bilayers on a chip and established conditions for incorporating these poly(C9) pores in these bilayers. Finally, we performed electrical characterization of the membrane-reconstituted pore and optimized the composition of the recording buffer.

We used SDS-PAGE (Supplementary Figure S1A), TEM (Figure 1A, Supplementary Figure S1B), and mass photometry (Supplementary Figure S1C) to verify proper formation of poly(C9) in solution. SDS-PAGE confirmed formation of high-molecular weight oligomers resistant to SDS-mediated degradation^38^ (Supplementary Figure S1A). TEM revealed the characteristic appearance of the poly(C9) ring (Figure 1A) with an average inner diameter of 11.2 ± 1.9 nm (mean ± SD, *N* = 205, Supplementary Figure S1B). Mass photometry demonstrated that the dominant poly(C9) population has an average molecular weight of 1.54 MDa, corresponding to an assembly of 22 monomers,^30,31,40^ as expected (Supplementary Figure S1C).

We formed planar lipid bilayers from 1,2-diphytanoyl-*sn*-glycero-3-phosphocholine directly on a recording chip. To insert poly(C9) into this membrane, we added 2 μL of the poly(C9)-amphipol solution into the cis compartment of the recording chip and monitored membrane incorporation of poly(C9) *via* electrical recordings performed with two Ag/AgCl electrodes placed on the opposite sides of the bilayer while applying a potential difference of −100 mV between them (Figure 1B). Since we recorded from four independent planar lipid bilayers with a nominal diameter of 50 μm simultaneously, we typically observed the first spontaneous insertion of poly(C9) within 5 min.

Figure 1C–F display examples of individual insertion events, where a single-step increase in current indicates the formation of a pore in the membrane. The absolute current through the open pore increased with the increase of the ionic strength of the recording buffer from 50 mM to 1 M NaCl (Figure 1C–F), leading to an approximately 6-fold increase in the ratio between open pore current and its standard deviation (Figure 1C,F, Supplementary Figure S2A,B). This increased signal-to-noise ratio is beneficial for detection of small proteins. To characterize the stability of poly(C9) nanopores, we analyzed the noise of the recorded current, based on its power spectral density (PSD) before and after insertion of the poly(C9) pore at different NaCl concentrations. Supplementary Figure S2C–F shows examples of the PSD, indicating that low-frequency noise (≤2 kHz) in the presence of the poly(C9) pore increased slightly with increasing ionic strength of the recording buffer. This frequency range in biological nanopores may originate from one or several of the following sources of noise: protonation noise, temporal conformational changes of the pore, thermal noise, shot noise, *etc*.^41^ On the other hand, high-frequency noise did not change significantly, indicating that there were no important additional sources of noise at high bandwidth. To maximize the signal-to-noise ratio, we carried out all experiments at 1 M NaCl, which is common in nanopore experiments.^42^

The time it took for poly(C9) to insert into the bilayer was, however, significantly longer at 1 M NaCl than at the lower ionic strengths we tested. To promote reconstitution of poly(C9) into phospholipid membranes in a recording electrolyte containing 1 M NaCl, we added 0.2 μM of amphipol to the recording buffer. Electrical measurements remained stable, and the signal-to-noise ratio did not change significantly in the presence of amphipol (compare Figure 1F, Supplementary Figures S2C–F and S3), indicating that the presence of amphipol in the recording buffer had no detectable effect on the planar lipid bilayers or poly(C9) pores.

To further characterize membrane reconstituted poly(C9), we obtained current–voltage (*I*–*V*) curves at different salt concentrations. In all tested conditions, the poly(C9) pore displayed ohmic behavior, consistent with a cylindrical pore lumen and the absence of current rectification. In addition, the pores were stable over a wide range of applied potentials from −150 to +150 mV (Figure 1G, Supplementary Figure S4).

The pore diameter measured by pore conductance (Supplementary Note 1, Supplementary Figure S5) deviated slightly from the poly(C9) inner diameter measured by cryo-EM^30,31^ (Figure 1H). The maximal deviation was observed at 1 M NaCl where the measured pore diameter was equal to 9.8 ± 2.1 nm (mean ± SD, *N* = 36), compared to ≈12 nm in the cryo-EM structure.^30,31^ The lateral pressure in the planar lipid bilayer^44^ may contribute to compression of the diameter of membrane-inserted poly(C9) pores.

### Nanopores from Poly(C9) Yield Accurate Estimates of the Volume and Shape of Single Proteins

**Figure 2:**
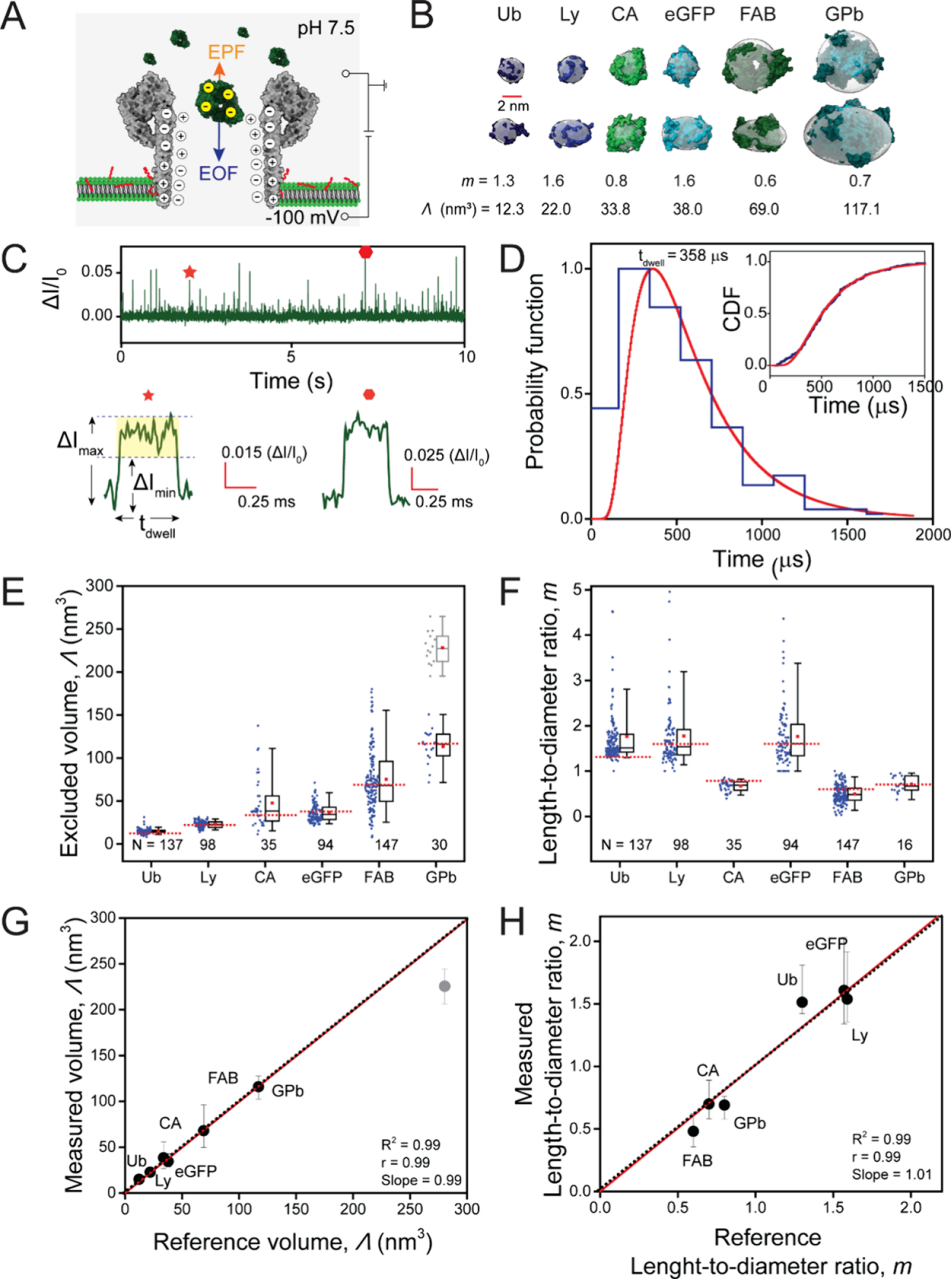
Estimation of the volume and length-to-diameter ratio of six natively folded proteins based on the resistive pulses in poly(C9) nanopores. (A) Schematic of a recording setup for single molecule analysis with poly(C9) pores embedded in a planar lipid bilayer. Arrows show the direction of electro-osmotic flow (EOF) and electrophoretic force (EPF) inside the pore at physiologic pH of 7.5. (B) Atomic structures of six test proteins and reference ellipsoids (in transparent gray) used to estimate their expected volume Λ and shape *m*. Dark blue–Ub, 1UBQ; blue–Ly, 1DPX; green–eGFP, 2Y0G; light blue–CA, 2CAB; dark green–FAB, 7FAB; and forest green–GPb, 1GPB (Supplementary Note 3). (C) Baseline–corrected current recording in the presence of FAB, showing resistive pulses as upward spikes. Recording buffer 1.0 M NaCl, 10 mM HEPES, pH 7.5, 0.2 μM amphipol, voltage = −100 mV applied to the bottom electrode. Current recordings shown here were collected with a 200 kHz sampling rate and filtered with 1 kHz Gaussian low-pass filter for clarity. Examples of two individual resistive pulses marked in the upper panel illustrate maximum current blockage (Δ*I*_max_), minimum current blockage (Δ*I*_min_), and dwell time (*t*_d_). (D) The distribution of dwell times *t*_d_ of resistive pulses from eGFP. Distribution includes the *t*_d_ values of all detected resistive pulses that were longer than 20 μs and shows that the most probable *t*_d_ value was 365 μs. The fraction of events exceeding the 300 μs threshold for approximation of the volume and shape of eGFP was 0.83. The inset shows the cumulative density function of experimentally measured dwell times. (E) Distributions of excluded volumes (Λ) and (F) length-to-diameter ratios (*m*) determined from individual resistive pulses obtained in the presence of one of the six test proteins in each experiment. Data for each protein was collected using three or four different pores; the average conductance and diameter of these pores are listed in Supplementary Table S1. Horizontal dotted red lines represent expected reference values, horizontal solid black lines represent median values experimentally determined with nanopore recordings. Solid red squares represent the experimental mean values. The box range represents 25th to 75th percentile and the whisker range displays the fifth to 95th percentile. (G, H) Median values of the excluded volume (Λ̅), and the length-to-diameter ratio (*m̅*) of test proteins plotted against the expected reference values. These proteins were tested individually. Error bars show the first and third quartiles. Black dotted lines represent the ideal 1:1 agreement (slope = 1), and the solid lines are the linear regressions performed imposing a zero intercept. Note that in E and G, the high-molecular-weight population in the GPb sample is shown in light gray. In G, we plotted this population against the volume (nm^3^) obtained by converting the molecular weight measured by mass-photometry^46^ (Supplementary Figure S11B).

To investigate the performance of the poly(C9) nanopores in determining the volume and shape of folded proteins, we added analyte proteins to the cis side of the pore while applying a constant potential of −100 mV to the bottom electrode across the lipid bilayer (Figure 2A). For the initial system characterization, we selected six proteins–ubiquitin (Ub, MW = 8.6 kDa, pI = 6.8), lysozyme (Ly, MW = 14.1 kDa, pI = 10.7), carbonic anhydrase (CA, MW = 30 kDa, pI = 6.6), enhanced green fluorescent protein (eGFP, MW = 33 kDa, pI = 6.8), human IgG Fab fragment (FAB, MW = 50 kDa, pI = 8.7), and glycogen phosphorylase b from rabbit muscle (GPb, MW = 97.2 kDa, pI = 7.2) (Figure 2B). Under the applied potential of −100 mV, resistive pulses in the current recordings indicate that all proteins were able to enter the poly(C9) pore (Figure 2C, Supplementary Figure S6).

Analysis of the event dwell time *t*_d_ revealed that the most probable dwell times for eGFP, Ly, and FAB were 358, 225, and 215 μs, respectively (Figure 2D, Supplementary Figure S7). The longest events for all proteins reached approximately 2 ms (Figure 2D, Supplementary Figure S7). These extended residence times facilitated accurate estimation of protein volume and shape, as each event provided a larger data set and sufficient time for the proteins to sample various orientations within the pore.

To determine the volume and shape of the selected proteins, we employed a theory developed by Golibersuch,^19,45^ which relates the amplitude of resistive pulses (Figure 2C, Supplementary Figure S8) to the volume of the particle and the current modulation during each resistive pulse to ellipsoid approximations of the particle’s shape. This theory does not account for a possible effect of the nanopore surface charge on its conductance, which is reasonable for a large-diameter nanopore and the NaCl concentration of 1.0 M used for all resistive pulse experiments with proteins. Based on this theory, we developed data analysis software described in detail in Supplementary Note 2. Importantly, the software continuously updated the calculated pore diameter based on the recorded open pore conductivity. This approach accounted for small dynamic changes in pore diameter, potentially arising from the insertion of an additional C9 monomer. As a result, we achieved consistent estimations of protein volume and shape (Figure 2E–H), regardless of variations in the specific pore diameter.

The automated algorithm detected individual resistive pulses for analysis if they met two criteria. First, the signal had to exceed a noise threshold level of five times the standard deviation of the open pore current (5σ). Second, proteins had to remain in the nanopore lumen sufficiently long to sample various orientations, including those that produce maximum and minimum current blockades (Δ*I*_min_ and Δ*I*_max_).^19,20^ We used a cutoff frequency of 10 kHz for the Gaussian low-pass filter to resolve resistive pulses from the noise for the smallest proteins (Ub and Ly). Consequently, we restricted the analysis of shape and volume of these proteins to resistive pulses lasting at least 450 μs. For larger proteins with molecular weights above 20 kDa (CA, eGFP, FAB, and GPb), a cutoff frequency of 20 kHz was sufficient to reach an adequate signal-to-noise ratio. Thus, for these proteins, we included resistive pulses lasting at least 300 μs for the analysis of shape and volume.^19,20^

To determine the volume and shape of proteins from resistive pulse recordings with high accuracy, it is crucial to know both the length and the diameter of the pore throughout the recording. Since pore conductance depends on both parameters, we assumed that the length of the poly(C9) pore remains constant and is, therefore, independent of the pore diameter. We then took an empirical approach to establish the effective poly(C9) pore length that resulted in the most accurate estimates of five different test proteins: plotting the volume of these proteins (Ub, Ly, CA, eGFP, FAB) determined from resistive pulses with poly(C9) pores as a function of their known reference volume (Supplementary Note 3) while assuming various pore lengths ranging from 10 to 17 nm. This analysis revealed that an effective pore length of 13 nm resulted in excellent agreement with a close-to-ideal slope of 1.06 ± 0.06 and a regression coefficient of *r* = 0.992 (Supplementary Figure S9). Therefore, we used a pore length of 13 nm for all further calculations of pore diameters from conductivity measurements. Moreover, we recalculated the pore diameter determined earlier based on the conductance of single poly(C9) pores with an assumed pore length of 16 nm (Figure 1H) using the updated effective pore length of 13 nm. The resulting updated values of pore diameters (Supplementary Figure S10) are only slightly smaller (<1 nm) than those reported in Figure 1H. However, they are likely more accurate in the context of nanopore experiments.

The measured excluded volume Λ and length-to-diameter ratio *m* for the test proteins agree well with the reference values derived from PDB structures (Figure 2E,F, Supplementary Note 3). Interestingly, the excluded volume Λ for GPb revealed two distinct populations in the protein sample. The first population had an average excluded volume of 116 ± 25 nm^3^, corresponding to a molecular weight of 95.7 ± 20.6 kDa.^46^ The second population exhibited an average Λ of 227 ± 21 nm^3^, corresponding to 187.3 ± 17.3 kDa. Mass photometry confirmed the presence of these two populations (Supplementary Figure S11). We attribute the first population to monomeric GPb, as both excluded volume Λ and length-to-diameter ratio *m* aligned well with the expected reference values of GPb (Figure 2E,F). The second population likely represents a GPb dimer^47^ since the determined volume is roughly double the volume determined for the GPb monomer, and the supplier specifies that the sample contains 5′-AMP, which promotes dimerization of GPb.^47^ Another possible reason for this second population could be the presence of impurities from other proteins in the sample. Indeed, the supplier specifies impurities by the following proteins in their GPb preparation: phosphorylase A (∼370 kDa), phosphoglucomutase (62 kDa), and phosphorylase kinase (1.3 MDa). However, since these three protein impurities differ significantly from the determined molecular weight of this second population of ∼190 kDa, we suggest that it likely results from dimers of GPb, which have been reported previously.^47^

Figure 2G,H show that the median estimates for the volume and length-to-diameter ratio deviated from the reference values by an average of only 4.8 and 3.0%, respectively (Supplementary Table S2). Note, the volume analysis includes all proteins, while the shape analysis includes all proteins except for the unidentified one because its reference shape is unknown. One reason for the excellent agreement of volume and shape estimates of all tested analyte proteins with their reference values is the exceptionally long dwell times (*t*_d_) observed in poly(C9) pores (Figure 2D, Supplementary Figure S7). With the most probable dwell times approximately 2 orders of magnitude longer than the diffusion time for a protein to traverse the nanopore, we propose that the ring-shaped distribution of opposing charges in its lumen (Figure 1B) may promote the formation of “electro-osmotic vortices”, as previously reported in PlyAB nanopores.^26^ These electro-osmotic vortices could transiently trap analyte proteins within the pore, thereby significantly increasing the dwell time of resistive pulse events.

An alternative explanation for the observed long residence times of analyte proteins in poly(C9) pores could be attractive interactions between the analyte proteins and the pore wall inside the lumen. Since the determined shapes of the tested proteins agree very well with their reference values, and since precise determination of shape requires sampling of the orientations that lead to determination of Δ*I*_min_ and Δ*I*_max_, we suggest that transient trapping of proteins by electro-osmotic vortices in the pore is more likely than sticking of proteins to the pore wall. Additionally, we never observed pore clogging, which further reduces the likelihood that binding interactions with the pore wall are responsible for the long residence times. While we cannot provide a definite mechanistic understanding of these favorably long resistance times at this point, the results in Figure 2 show that poly(C9) pores enable exceptionally accurate estimates of the volume and shape of analyte proteins with molecular weights up to 200 kDa.

### Resolving Individual Proteins in a Mixture of Proteins

**Figure 3:**
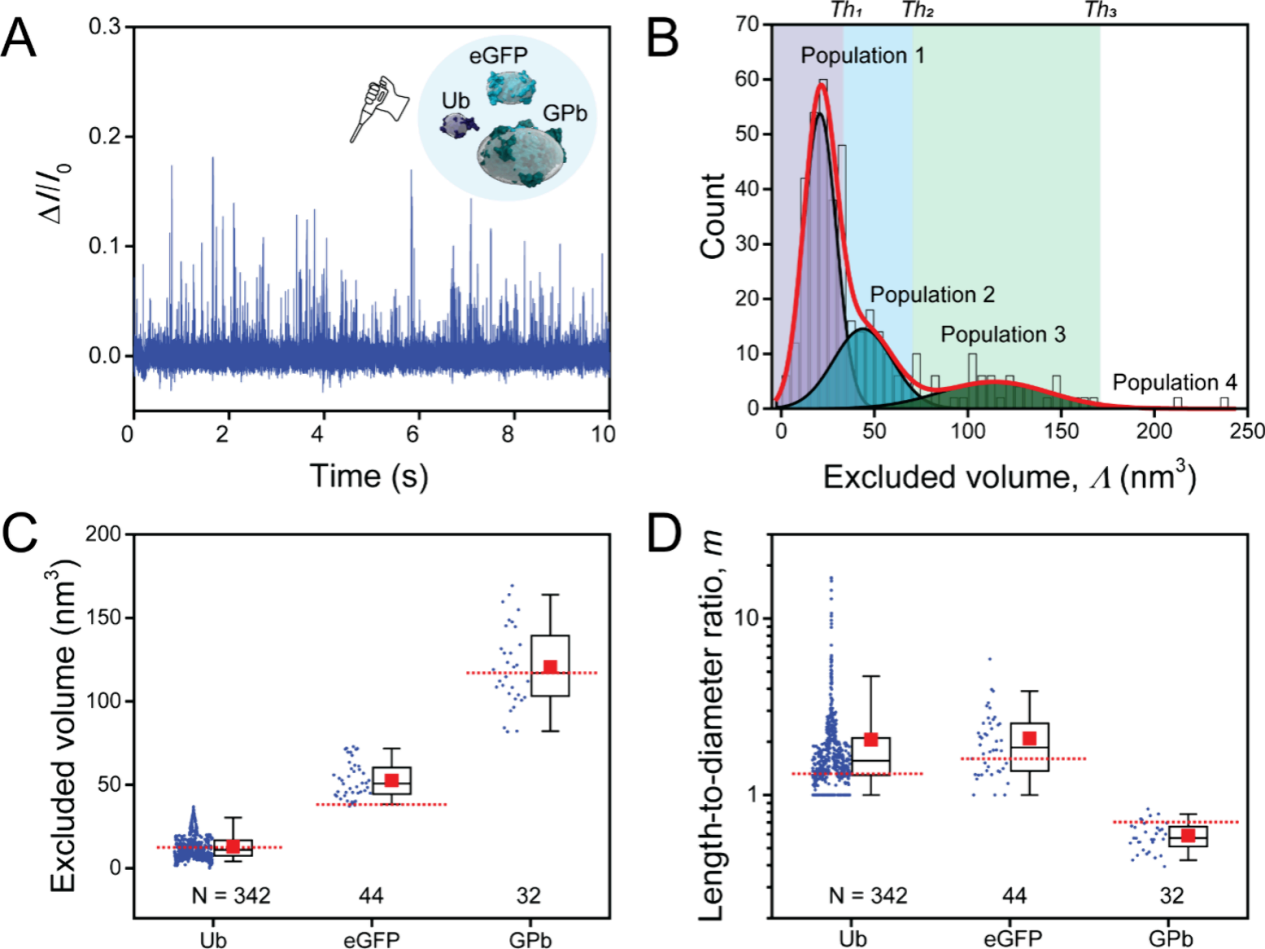
Analysis of a mixture of three proteins using poly(C9) nanopores. (A) Baseline–corrected current recording of an equimolar mixture of Ub, eGFP, and GPb, showing resistive pulses as upward spikes. Recording buffer 1 M NaCl, 10 mM HEPES, pH 7.5, 0.2 μM amphipol, voltage = −100 mV applied to the bottom electrode. The current recordings were collected with a 200 kHz sampling rate and filtered with a 10 kHz Gaussian low-pass filter for clarity. (B) Histogram of excluded volume distribution from the protein mixture. Thresholds (*Th*_*i*_) used to separate the data into distinct protein populations are indicated on top of the graph and by shaded regions. (C) Distribution of excluded volumes (Λ) and (D) length-to-diameter ratios (*m*) determined from individual resistive pulses obtained in the protein mixture and separated for data analysis by thresholding the histogram as shown in B. Data was collected using three different pores; the average conductance and diameter of these pores are listed in Supplementary Table S1. Horizontal dotted red lines represent expected reference values, horizontal solid black lines represent median values experimentally determined with nanopore recordings. Solid red squares represent the experimental mean values. Box range represents 25th to 75th percentile and whisker range displays fifth to 95th percentile.

To test the ability of the poly(C9) nanopores to detect individual proteins within a mixture of proteins, we prepared a 1:1:1 molar mixture of Ub, eGFP, and GPb and recorded the resulting resistive pulses (Figure 3A). Single-molecule analysis revealed a polymodal distribution of excluded volumes, Λ (Figure 3B). To assign resistive pulses to specific protein populations, we fitted the volume distribution with three Gaussian peaks and established thresholds between the peaks. To minimize bias, thresholds (*Th*) were set on the right side of each peak at *Th*_*i*_ = μ_*i*_ + σ_*i*_, where μ is the mean value of peak *i* = (1, 2, 3), and σ is its standard deviation. The thresholding separated four protein populations corresponding to Ub, eGFP, GPb monomer, and GPb dimer (Figure 3B). The GPb dimer population, consisting of only a small number of events, was excluded from further analysis.

Subsequently, we analyzed the three main groups of resistive pulses individually for their volume and shape. The resulting box plots in Figure 3C,D demonstrate good agreement with the reference values for all three proteins (Supplementary Table S3). Note, the shape analysis incorporated a priori information about the prolate or oblate geometry of each protein.

### Poly(C9) Nanopores Resolve Different Conformations of Adenylate Kinase

**Figure 4:**
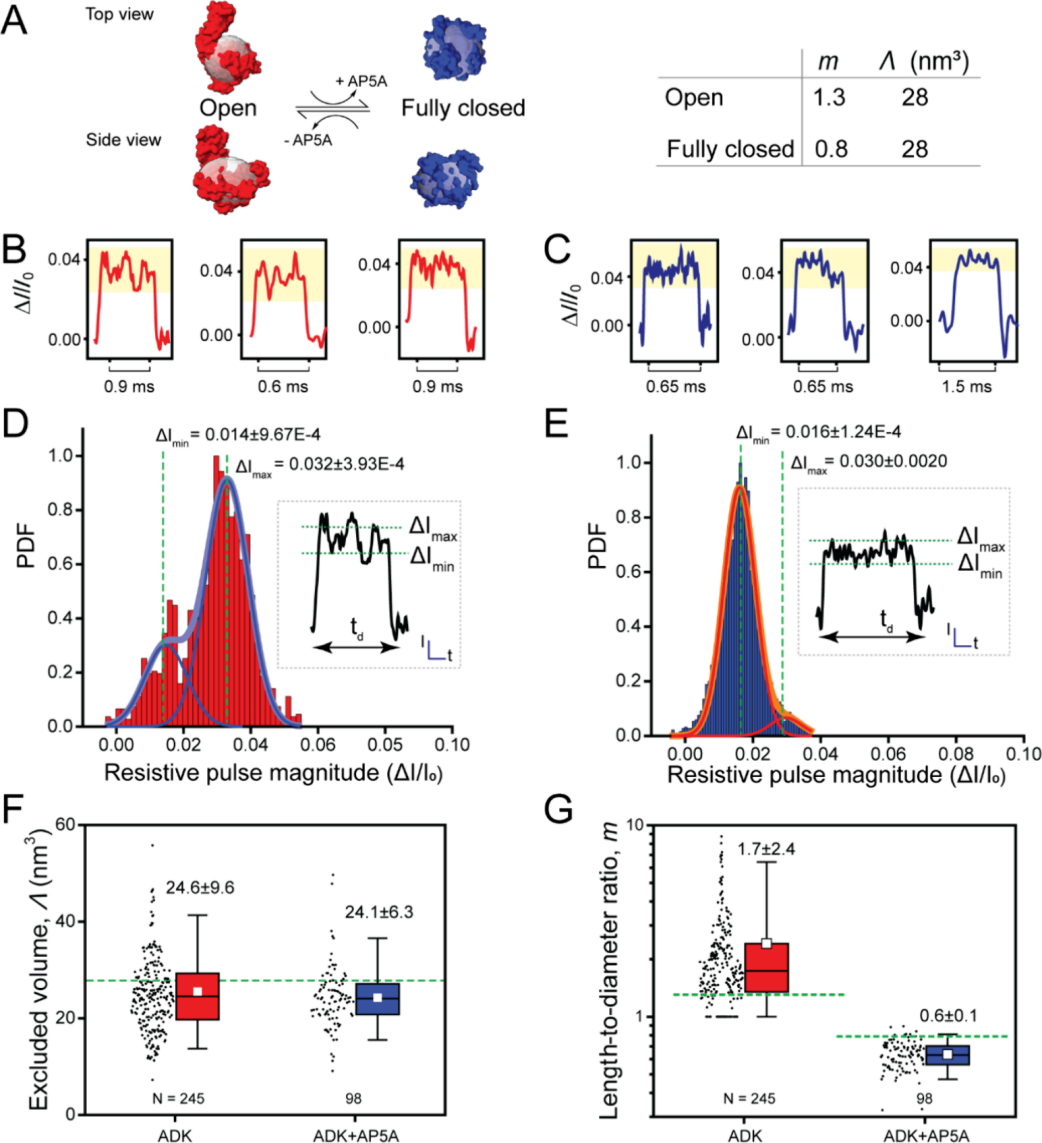
Application of poly(C9) nanopores for resolving the open and closed conformation of ADK. (A) Schematic illustration of the conformational transition from open apo ADK (PDB 4AKE) to fully closed holo ADK (PDB 1AKE) upon binding the inhibitor AP5A. Reference ellipsoids used to estimate protein volume and shape are shown in transparent gray. (B, C) Representative individual resistive pulses from ADK in the absence (B, red) and in the presence of AP5A (C, blue). Current modulation is highlighted in yellow. (D, E) Histograms of relative resistive pulse magnitude of ADK in the absence of AP5A (D, red, *N* = 68) and in the presence of AP5A (E, blue, *N* = 82). Insets display typical resistive pulses. (F) Distribution of excluded volumes (Λ) and (G) length-to-diameter ratios (*m*) determined from individual resistive pulses obtained from ADK in the absence of AP5A (red) or presence of AP5A (blue). Data was collected using three or five different pores; the average conductance and diameter of the pores are listed in Supplementary Table S1. Horizontal dotted green lines represent expected reference values, and horizontal solid black lines represent median values experimentally determined with nanopore recordings. Solid white squares represent the experimental mean values. Box range represents 25th to 75th percentile and whisker range displays fifth to 95th percentile.

To test the ability of the poly(C9) nanopore to distinguish proteins by shape and volume, we employed a well-studied model protein, adenylate kinase (ADK, MW = 23.7 kDa) to explore if resistive pulses using poly(C9) pores can resolve the open and closed conformation of this enzyme. ADK plays a crucial role in cellular energy metabolism by catalyzing the reversible phosphoryl transfer between adenine nucleotides, primarily ATP and AMP, leading to the production of ADP.^48−50^ During its catalytic cycle, ADK undergoes a substantial conformational change. In the apo state, ADK adopts an open conformation with accessible binding sites; this open conformation is dominant in physiologic solution in the absence of substrates or inhibitors. In the holo state, ADK closes to minimize unproductive active site fluctuations and nonproductive hydrolysis.^51,52^ When exposed to multisubstrate inhibitors such as diadenosine pentaphosphate (AP5A), ADK adopts a fully closed conformation.^53^ Conformational changes in ADK upon binding to AP5A do not affect the total volume of the protein but alter its length-to-diameter ratio (Figure 4A). Here we tested if poly(C9) nanopores make it possible to discriminate between apo and holo conformations of ADK.

We added ADK (1.4 μM) with or without AP5A (140 μM) to the cis compartment of the recording chip. Under a negative applied potential of −100 mV (to the trans electrode) at 1 M NaCl, 2 mM MgSO_4_, 0.2 μM amphipol, and 10 mM HEPES (pH = 7.5), we observed individual resistive pulses (Figure 4B,C) and plotted histograms of all current modulation values (Figure 4D,E). The first thing we tested on these histograms is if they were distributed normally or if they were significantly different from a Normal distribution. For a perfectly spherical protein with an *m* value of 1.0, the distribution would be Normal as we have confirmed previously.^19,20^ If the Δ*I*/*I*_0_ distributions were significantly different from Normal, we attributed this deviation from Normality to an ellipsoidal shape of the protein, which leads to two extremes of Δ*I*/*I*_0_ values (Δ*I*_min_/*I*_0_ and Δ*I*_max_/*I*_0_) depending on the orientation of the ellipsoid inside the electric field in the nanopore. In this case, we fitted the distributions with two Gaussian peaks to determine Δ*I*_min_/*I*_0_ and Δ*I*_max_/*I*_0_, which then yield the *m* value of an ellipsoid that most closely matches the 3-D structure of the protein. The first peak at 0.014 ± 0.0097 for apo ADK (Figure 4D) and 0.016 ± 0.0001 for holo ADK (Figure 4E) corresponds to the minimal current blockage. The second peak at 0.032 ± 0.0004 for apo ADK (Figure 4D) and 0.030 ± 0.0020 for holo ADK (Figure 4E) corresponds to the maximum current blockage. The distribution of the data between those two peaks reflects the distribution of particle orientations, which depends mainly on its shape. Because of the symmetry of an ellipsoid of revolution (with axes: A, B, B), these particles exhibit a higher probability to be oriented with the symmetry axis perpendicular to the nanopore axis (Supplementary Note S4). As a result, the Δ*I*/*I*_0_ distribution of prolate ellipsoids is strongly skewed toward Δ*I*_max_/*I*_0_, while that of oblate ellipsoids is strongly skewed toward Δ*I*_min_/*I*_0_. This difference implies that the ratio between the mean and the median of the Δ*I*/*I*_0_ distribution makes it possible to distinguish between a prolate (mean/median >1) and an oblate (mean/median <1) ellipsoid. Specifically, for apo ADK, the maximum blockage dominated, indicating that the apo conformation of ADK is best described by a prolate shape. In contrast, for holo ADK, the minimum blockage dominated the histogram, suggesting that the holo conformation of ADK is best described by an oblate shape.

We next measured the excluded volume Λ and length-to-diameter ratio *m* for apo and holo ADK. The excluded volume in both conformations was similar and in excellent agreement with the reference values based on PDB structures: 24.6 ± 9.6 nm^3^ in the open apo conformation and 24.1 ± 6.3 nm^3^ in the closed holo conformation (Figure 4F). On the other hand, the length-to-diameter ratios were significantly different, making it possible to distinguish between the two conformations of ADK: 1.7 ± 2.4 in the open apo conformation and 0.6 ± 0.1 in the closed holo conformation (Figure 4G). Both values were in good agreement with the length-to-diameter ratio of the two conformations based on PDB structures (Figure 4G).

In summary, poly(C9) nanopores proved to be effective in discriminating proteins with the same volume by their conformation.

## Discussion

### Limitations of Poly(C9) Nanopore Sensing Devices

Regarding the upper size limit of proteins that can be characterized on a single particle level with poly(C9) pores, it was straightforward to determine the shape and volume of GPb with a molecular weight of 97 kDa with good agreement with reference values. We also detected an unknown protein species whose determined size agreed reasonably well with the one revealed by mass photometry (∼230 kDa, Figure 2G). In any case, the upper limit of accessible protein sizes for characterization with poly(C9) pores expands the 80 kDa limit observed previously with PlyAB nanopores^26^ and is presumably due to the almost double diameter of the poly(C9) pore compared to the PlyAB pore. The smallest protein characterized in this study is Ub (MW = 8.6 kDa), and preliminary data suggests that proteins with MW ≈ 5 kDa could also be detected and analyzed. Future studies are needed to systematically establish the upper and lower molecular weight limits for detection with poly(C9) nanopores.

An inherent challenge with biological nanopores is the stability of the supporting membrane and the pores under applied voltages, particularly beyond 300 mV.^54^ We started to observe instability in the membrane at applied potential differences above |±100 mV| and the duration of experiments was typically limited by the irreversible breakdown of the planar lipid bilayer membrane rather than a detectable degradation of the poly(C9) pores (see Supplementary Figure S12). While this voltage limitation did not impede our ability to gather structural information about analyte proteins, it could pose challenges in future applications. One potential solution would be to explore the use of artificial copolymer membranes (e.g., diblock copolymer poly(1,2-butadiene)-*b*-poly(ethylene oxide), PBD–PEO^54^) that may provide greater stability under higher voltage conditions.

The preparation of poly(C9)-amphipol is not uniform, as it contains a mixture of oligomers, including not only the desired ring structures with 22 ± 4 monomers but also a significant fraction of much smaller, incomplete assemblies of 2–9 monomers of C9 as revealed by mass photometry data (Supplementary Figure S1C). Despite this heterogeneity, recording the amplitude of the open pore current makes it possible to update the pore diameter during resistive pulse recordings and hence to account for variability in pore diameter from experiment to experiment as well as during individual recordings; all analyses in this work used continuously determined open pore current values to determine the pore diameter. These measurements of pore diameter make it possible to proceed only with experiments with pores of desired size while terminating those with pores that are too small or too large. Future improvements could involve separating various sizes of poly(C9) pores, possibly preceded by cross-linking^55^ of the pores to stabilize their sizes during separation and storage.^52^

### Comparison of Poly(C9) and Solid-State Nanopore Sensor Devices

When comparing poly(C9) nanopores to solid-state nanopores in their ability to analyze folded proteins by volume and shape, the results presented here indicate significantly improved (*i.e.*, lower) uncertainty in determined volumes and shapes. Notably, in this study, we observed average deviations from reference values for volume and for length-to-diameter ratio below 5% (Supplementary Table S2). This level of accuracy represents a substantial improvement over previous results with solid-state nanopores, which showed average deviations around 20%.^19^ This comparison underscores the reliability of the poly(C9) nanopores in producing consistent measurements for protein characterization in a range of molecular weights from 10 to at least 100 kDa, which covers more than half of all distinct human proteins.^56,57^ Presumed reasons for this benefit are the constant and hence reproducible effective length of poly(C9) nanopores as well as their predominantly cylindrical lumen. There is a slight narrowing (≈ 1 nm) at the HTH region at the entrance to the pore (Figure 1B), but it appears to be accounted for by determination of the effective pore length.

Another illustrative example of the accuracy of poly(C9) nanopores is provided by the results from characterization of ADK. Previously, ADK has been used to investigate possible differences between the apo and holo state of ADK with respect to their transport through narrow solid state nanopores with a diameter of 7 nm.^58^ In that study, the size of the nanopore was sufficiently small that ADK was squeezed through the pore by a pressure difference. Under these conditions, the authors were able to distinguish the apo and holo state of ADK based on detectable differences in capture rates, drift velocities, and diffusion coefficients measured with these narrow solid-state nanopores. The work presented here takes a different approach by employing a pore with a diameter that is at least 5 nm larger than the longest dimension of ADK, allowing the enzyme to rotate within the pore. This method made it possible to determine the difference in shape of two conformations of ADK in its physiological state. This capability highlights the versatility of nanopore technologies and sets a precedent for examining native protein conformations in poly(C9) pores and possibly other large biological nanopores in the future.

## Conclusions

In summary, the results presented here demonstrate that poly(C9) pores, self-assembled in solution in the presence of amphipol, could be easily inserted into a planar lipid bilayer on a nanopore chip for resistive pulse experiments. The diameter and shape of the poly(C9) nanopore lumen allow analyte proteins with molecular weights ranging from 9 to 230 kDa to enter the pore, generating resistive pulse signals that enable accurate determination of analyte volume and shape. Therefore, the poly(C9) nanopore holds the potential for advancing our understanding of the conformational dynamics of large proteins and enzymes at the single-molecule level.

### Methods

#### Assembly of Complement Component 9 (C9)

We assembled poly(C9) rings from monomers as previously described.^30,31^ Briefly, we concentrated a 1 mg/mL monomeric solution of human C9 (Sigma-Aldrich, cat. 204910) *via* a buffer exchange procedure using a 30 kDa MWCO protein spin filter (Amicon) to obtain a final concentration of 2 mg/mL in a buffer containing 10 mM HEPES pH 7.5, 50 mM NaCl (poly(C9) buffer). We prepared a 2 μM stock solution of amphipol A8-35 (Anatrace, cat. A8-3535) by resuspending the dry powder in poly(C9) buffer. Monomeric C9 and amphipol were mixed in a 128:1 molar ratio. We aliquoted this mixture in 10 μL volumes and incubated them overnight at 37 °C.^30,31^ After incubation, we kept the poly(C9)-amphipol sample at 4 °C and used it for experiments within seven days.

#### Formation of Lipid Bilayers on a Chip

Lipid bilayers were formed on a four-well MECA recording chip with 50 μm cavity size (Ionera, cat. 132001) using an Orbit mini from Nanion Technologies equipped with a four-channel low-noise amplifier from Elements SRL. To form planar lipid bilayers, we followed the technique described by Wang *et al.*(59) Briefly, 150 μL of the recording buffer containing 10 mM HEPES pH 7.5, and NaCl concentrations ranging from 50 mM to 1 M was filtered with a 0.2 μm poly(ether sulfone) filters (VWR, Radnor, PA, USA) and added to the cis compartment of the clean MECA chip. For the 1 M NaCl condition, 0.2 μM amphipol was added to the recording buffer. In order to displace trapped air from the cavities in the MECA chips and fill them with recording electrolyte, we applied a gentle pressure with a syringe plunger. The bilayers were then manually formed from a 15 mg/mL solution of 1,2-diphytanoyl-*sn*-glycero-3-phosphocholine (Sigma-Aldrich, cat. 850356P) in *n*-octane (Sigma-Aldrich, cat. 296988) as follows. A pipet was set at 1.5 μL and a tip was dipped into the lipid solution without pipetting. The tip was then carefully dried with a tissue paper, leaving only a trace amount of lipids on the tip surface. Next, it was placed in the vicinity of a cavity on the chip and pipetted once to make an air bubble on the cavity. Once the lipid bilayer was formed, we aspirated the air bubble from the cavity. To confirm formation of the membrane, a baseline current (−0.3 < *I* < 0.3 nA) and capacitance (7 ± 2 nF) were measured using EDR5 software (Elements). The stability of the bilayer (absence of leak currents, noise level of approximately 30 pA rms at full bandwidth of ∼100 kHz) was verified by applying transmembrane voltages ±100 mV for 1 min before adding poly(C9).

After this preparation of planar lipid bilayers, the MECA chip was ready for poly(C9) insertion into the membrane.

#### Membrane Insertion of Poly(C9) and Initial Characterization of the Nanopore

Two microliters of the poly(C9)-amphipol solution was added into the cis compartment of the MECA recording chip to obtain a final concentration of C9 of 92 nM (monomer equivalent). An applied potential of +100 mV was maintained until a single-step jump in the measured current was observed, indicating incorporation of a poly(C9) pore into the lipid bilayer. If we did not observe pore insertion in 5 min, we broke the membranes with a high voltage pulse and restarted the experiment by forming new membranes.

The diameter of the pore was estimated using an equation proposed by Cruickshank *et al.*,^60^ assuming the pore to be perfectly cylindrical**:**
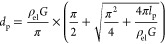
1

In eq 1, *d*_p_ (m) is the inner diameter of the nanopore, *l*_p_ (m) is the effective pore length empirically estimated as 13 nm (Supplementary Figure S9), ρ (Ω·m) is the electrical resistivity of the electrolyte buffer and *G* (S) is the conductance of the pore. This equation accounts for the electrical resistance of the pore as well as the access resistance to the pore on either side of it.

We proceeded with resistive pulse recordings only if the estimated pore diameter was between 5 and 15.5 nm (corresponding to an open pore current between 1 nA and 6.5 nA at an applied potential difference of +100 mV), which was the case in ∼80% of experiments. Significant changes in the open pore current during resistive pulse recordings (>1 nA at ±100 mV) were interpreted as the insertion of a second pore, and such measurements were discontinued.

The number of C9 monomers composing the nanopore was determined using a previously published geometrical model^61^ (Supplementary Note 1).

#### Planar Lipid Bilayer Recordings

Electrical recordings were carried out using an integrated, chip-based, four-channel, parallel bilayer recording setup (Orbit Mini, Nanion Technologies) and a EDR5 software (Elements). The top (cis) compartment on the MECA recording chip was connected electrically to the ground, while the four (trans) compartments underneath each of the four lipid membranes, contained Ag/AgCl working electrodes; each of these electrodes could separately apply a different desired command voltage relative to the common ground. All measurements were performed at 25 °C with a sampling rate of 200 kHz for each one of the four recording channels and a current range of 20 nA. Current traces were filtered with a Gaussian low-pass filter with a cutoff frequency of 20 kHz or 10 kHz and analyzed using Clampfit 11.2 (Molecular Devices) and an in-house Python script developed to analyze protein volume and shape (Supplementary Note 2 and Supplementary Figure S13).

#### Protein Resistive Pulse Sensing Experiments

After insertion and initial characterization of a poly(C9) pore, a stepwise potential sweep from −100 to +100 mV was performed to verify pore stability and measure the current offset at 0 mV applied potential. Subsequently, 5 μL solution containing 1 mg/mL test protein was added into the common cis compartment of the MECA recording chip. Resistive pulses of the proteins were promoted by applying a constant potential of either +100 or −100 mV across the planar lipid bilayer with inserted nanopore. The potential sweep was repeated every 5 min, to verify the stability of the nanopore and determine possible changes in offset current. We took this offset current into account during the pore diameter calculation and the analysis of resistive pulses to increase the accuracy of our measurements.

We tested the resistive pulse sensing of seven different proteins: human ubiquitin (Ub, Sigma-Aldrich, cat. U5507), lysozyme from chicken egg white (Ly, Sigma-Aldrich, cat. L6876), enhanced green fluorescent protein (eGFP, His tagged, Abcam, cat. ab51992), carbonic anhydrase I from human erythrocytes (CA, Sigma-Aldrich, cat. C4396), human IgG Fab fragment (FAB, Rockland, cat. 009-0105), adenylate kinase (ADK, Sigma-Aldrich, cat. SRP6121) with or without an inhibitor P^1^,P^5^-Di(adenosin-5′) pentaphosphate pentasodium salt (AP5A, Sigma-Aldrich, cat. D4022), and glycogen phosphorylase b from rabbit muscle (GPb, Sigma-Aldrich, cat. P6635). Final molar concentrations of analyte proteins in the cis compartment were equal to 116 μM for Ub, 71 μM for Ly, 27 μM for eGFP, 33 μM for CA, 20 μM for FAB, 10.3 μM for GPb, and 1.4 μM for ADK.

#### Gel Electrophoresis

Samples were mixed with 4x LDS sample loading buffer (nonreducing, Thermo Scientific, cat. 84788) before adding them to the wells. Electrophoresis was carried out on 4–15% Mini-PROTEAN TGX Precast Protein Gels (Bio-Rad, cat. 4561086) under an applied potential of +250 mV for 40 min. Subsequently, the gel was stained overnight with ReadyBlue Protein Gel Stain (Agilent, cat. RSB-1L). We used a wide range SigmaMarker (Sigma, cat. S8445) to estimate protein molecular weight on the gel.

#### Transmission Electron Microscopy (TEM)

We used a fresh glow discharged 300 mesh carbon-coated copper grid (Thermo Fisher Scientific, cat. 5024892) for all the TEM measurements. A 3 μL droplet of poly(C9)-amphipol sample (∼2 mg/mL) was incubated on a grid for 30 s before adding 3 μL of 2% v/v aqueous uranyl acetate solution and incubating for another 2 min. The grid was then blotted with a filter paper and dried at room temperature for 30 min. The TEM imaging was performed using a Tecnai G2 electron microscope (FEI) at 120 kV. We used ImageJ^62^ to analyze TEM data.

#### Mass Photometry

We acquired mass photometry data with a TwoMP mass photometer (Refeyn Ltd.) using a protocol described by Kukura *et al*.^63^ with slight modifications. Microscope coverslips (24 × 50 mm Thorlabs, cat. CG15KH1) and self-adhesive silicone wells (Sigma-Aldrich, cat. GBL103250) were used for the measurements. We mixed a volume of 2 μL of a 0.2 mg/mL solution of poly(C9)-amphipol with 18 μL of poly(C9) buffer in a well. For GPb experiments, we used 0.01 mg/mL protein solution and either poly(C9) recording buffer with 1 M NaCl pH 7.5 or PBS pH 7.4. Mass photometry recording was carried out for 180 s. Contrast-to-mass calibration was performed using NativeMark Unstained Protein Standard (ThermoFisher SCIENTIFIC, LC0725). The data was analyzed using DiscoverMP software.

#### Handling of Atomic Structure

We used ChimeraX^64^ to visualize the atomic structures of proteins, and we estimated the electrostatic potential of the poly(C9) pore using the APBS calculation^65^ for two adjacent C9 monomers.

## Supporting Information Available

Details of estimation of pore inner diameter and number of monomers, details of data analysis algorithm, details of approximation of protein shape with an ellipsoid of revolution, details of impact of ellipsoid shape on the Δ*I*/*I*_0_ distribution, characterization of poly(C9)-amphipol oligomers in the solution, effect of NaCl concentration in the recording buffer on the current noise from recordings with a single poly(C9) pore, characterization of a single poly(C9) nanopore in the recording buffer with 1 M NaCl in the absence of amphipol, current–voltage (*I*–*V*) curves of the poly(C9) pore from −150 to +150 mV, conductance distributions of poly(C9) pores in planar lipid bilayers, baseline–corrected current recordings showing resistive pulses (upward spikes) measured with poly(C9) nanopores, the distribution of dwell times *t*_d_ of resistive pulses, representative individual resistive pulses, empirical determination of the effective pore length of poly(C9) pores, estimations of the effective inner diameter of the poly(C9) pore at different NaCl concentrations, size distribution of a sample containing commercially available GPb by mass photometry recording in two different buffers, termination of the experiment due to membrane rapture, schematic representation of the nanopore data analysis, details of the number, conductance, and diameter of poly(C9) nanopores used in resistive pulse experiments, details of excluded volumes and length-to-diameter ratios determined by analysis of resistive pulses and their deviations from the reference values, and excluded volumes and length-to-diameter ratios determined by analysis of resistive pulses of three mixed proteins compared to their reference values (PDF)
