# Supplementary material for: Large and Stable Nanopores Formed by Complement Component 9 for Characterizing Single Folded Proteins
Source: ACS Nano. 2025 Jan 28;19(5):5240–52. doi: 10.1021/acsnano.4c11666 (PMC11823641; doi:10.1021/acsnano.4c11666)
Supplement: Supplementary file 1 — nn4c11666_si_002.pdf [file nn4c11666_si_002.pdf]

Supporting Information

Large and stable nanopores formed by complement component 9 for characterizing single folded proteins

Wachara Chanakul,[1] Anasua Mukhopadhyay,[1,2] Saurabh Awasthi,[1,3] Anna D. Protopopova,[1] Alessandro Ianiro,[1,2,4] Michael Mayer[1,2,*]

[1] Adolphe Merkle Institute, University of Fribourg, Fribourg 1700, Switzerland

[2] National Center for Competence in Research Bio-inspired Materials, University of Fribourg, Fribourg 1700, Switzerland

[3] Present Address: Department of Biotechnology, National Institute of Pharmaceutical Education and Research Raebareli (NIPER-R), Lucknow 226002, UP, India

[4] Present Address: Department of Chemistry, KU Leuven, Leuven 3001, Belgium

* Corresponding Author Email ID: michael.mayer@unifr.ch

Table of Contents

Supplementary Note 1: Estimation of pore inner diameter and number of monomers 2

Supplementary Note 2: Data analysis algorithm 3

Supplementary Note 3: Approximation of protein shape with an ellipsoid of rotation 5

Supplementary Note 4: Impact of ellipsoid shape on the □I/I_0 distribution 5

Supplementary Figures 7

Supplementary Tables..21

References..24

Supplementary Note 1: Estimation of pore inner diameter and number of monomers

We calculated the equivalent poly(C9) pore diameter based on its conductance by comparing the baseline current with the current after single-pore insertion. We used the equation presented by Cruickshank *et al.*:[1]

$$d_\mathrm{p} = \frac{\rho_\mathrm{el}\,G}{\pi} \times \left(\frac{\pi}{2} + \sqrt{\frac{\pi^2}{4} + \frac{4\pi l_\mathrm{p}}{\rho_\mathrm{el}\,G}}\right) \tag{1}$$

In this equation:

d_p (m) — Inner diameter of the nanopore,

ρ_el ($\Omega \cdot \mathrm{m}$) — Electrical resistivity of the electrolyte buffer, we used 8.63 $\Omega \cdot \mathrm{m}$ for the recording buffer with 1 M NaCl,

G (Ω^{-1}) — Conductance, determined as the ratio of the difference between the baseline current and the open-state pore current at the applied voltage,

l_p (m) — Effective pore length was estimated to be 13 nm (see Supplementary Figure S8) and assumed to be constant for all pore diameters.

We then used the determined pore diameter to estimate the number of C9 monomers comprising the pore using a geometric model described by Fennouri *et al.*:[2]

$$d_p = s\sqrt{\frac{1}{\pi}\left(\frac{n}{tan\left(\frac{\pi}{n}\right)} - \frac{(n-2)\pi}{2}\right)} \tag{2}$$

In this equation:

d_p (m) — Inner diameter of the nanopore, obtained from Equation (1),

n — Number of monomers in the poly(C9) pore,

s (m) — Diameter of a cylindrical rod representing a C9 monomer.

The model assumes that the poly(C9) pore is a circular arrangement of cylindrical rods (C9 monomers) oriented perpendicular to the surface of a planar lipid bilayer. These rods are positioned at the corner points of a regular polygon, with the diameter of each rod equal to the distance between the rods.

We have previously demonstrated[2] that a Taylor expansion of Equation (2) provides an accurate estimation of pore diameter:

$$d \approx s(0.318n - 0.784) \tag{3}$$

To determine the diameter s of a cylindrical C9 monomer, we used Equation (3) and information from a recent cryo-EM structure[3–5] where the inner diameter of poly(C9) was found to be 12 nm, and the pore consists of 22 monomers. Consequently, the diameter of a cylindrical rod representing a C9 monomer in poly(C9) is approximately 1.93 nm.

Supplementary Note 2: Data analysis algorithm

We developed data analysis software to obtain excluded volume Λ and length-to-diameter ratio m of a target analyte from nanopore recordings. The software is schematically depicted in Supplementary Figure S12 and detailed below.

The analysis consists of three sequential steps:

1. Baseline search. In the first step, the recording x, composed of W samples is imported and digitally filtered using a Gaussian low-pass filter with a desired cutoff frequency. The data are subsequently processed with a custom-made baseline search algorithm that operates as follows:

1) the filtered recording x^{f}, with size W, is subdivided into $N+1$ segments, N containing M samples each and one containing the remaining $W-NM$ samples. The value of M is set by the user and should always be M >100 and at least 30 times longer than the longest translocation event.
2) An empty array x^{b} with length W is initialized, which will be filled with the estimated baseline values. For clarity, the jth element of an array x is indicated as $x[j]$.
3) The mean ($\bar{y}_{\mathrm{i}}$) and the standard deviation (σ_{i}) of each segment y_i is calculated.
4) The minimum standard deviation $\sigma_{\min}$is identified as the minimum value among the standard deviations of the M-sized segments y_i.
5) A threshold parameter $\tau > 1$ is defined (typically 1.2< τ <1.6).
6) For each segment y_i, with $i = 1 \ldots N$, the standard deviation σ_{i} is compared to $\tau\sigma_{\min}$ with two possible outcomes:
   A) if $\sigma_{\mathrm{i}} \leq \tau\sigma_{\min}$, the elements of x^{b} comprised between $x^{\mathrm{b}}[(i-1)M]$ and $x^{\mathrm{b}}[iM]$ are filled with the average value $\bar{y}_{\mathrm{i}}$
   B) if $\sigma_{\mathrm{i}} > \tau\sigma_{\min}$, y_i is subdivided into 10 samples, z_k^i ($k = 1 \ldots 10$) of length M/10. The elements of x^{b} comprised between $x^{\mathrm{b}}[(i-1)M + M(k-1)/10]$ and $x^{\mathrm{b}}[(i-1)M + Mk/10]$ are filled with the average value of the z_k^i segment, $\bar{z}_k^i$.
7) The baseline is finally completed by filling the last $W-NM$ elements of x^{b} with the mean value of the last $W-NM$ elements of x^{f}.

The baseline trace x^{b} is important because it allows tracking step changes in the baseline current and, hence, changes in the diameter of the nanopore over time.

2. Event detection. After determining the baseline trace, the normalized ($\Delta I/I_0$) trace, x^{n} is calculated as $x^{\mathrm{n}} = (x^{\mathrm{f}} - x^{\mathrm{b}})/x^{\mathrm{b}}$ and processed with the following threshold-based event detection algorithm:

1) The trace x^{n} is subdivided into $F + 1$ segments, F containing G samples each and one containing the remaining $W - FG$ samples.
2) For each G-sized segment q_i, with $i = 1 \dots F$, the mean $\bar{q}_i$ and the standard deviation σ_i are computed. Two thresholds are calculated, $T_{\mathrm{s}} = \bar{q} + u\sigma_i$, where $u > 1$ (typically $u > 4$), and $T_{\mathrm{e}} = \bar{q} + \sigma_i$.
3) A preliminary search is performed to identify at which position in q_i the signal first exceeds T_{s} (e_{s}, event start) and then falls below T_{e} (e_e, event end). For each event, the end and start position of the events in the trace x^{n} are calculated to include part of the baseline as $E_{\mathrm{s}} = e_{\mathrm{s}} + (i-1)G - 40$ and $E_{\mathrm{e}} = e_{\mathrm{e}} + (i-1)G + 40$, and stored (extended events).
4) The detected events are subsequently refined, as a simple threshold-based search could fragment a single translocation into multiple short events, or interpret noise spikes as translocation events:
   A) All samples in x^{n} with value smaller than T_{s} are stored in an array w of size R.
   B) A matrix $B_{(R-Q)\times Q}$ is constructed by stacking Q delayed copies of w, such that the jth column of *B* contains the elements of w comprised between $w[j]$ and $w[R-Q+j]$.
   C) The standard deviation of each row of *B* is calculated, and the minimum value obtained is defined σ_0.
   D) Each extended event is fitted using two Gaussian peaks having the same standard deviation σ_0 and centered at positions L_0 and $L_1 (L_1 > L_0)$. L_0 and L_1 represent an estimate of the baseline and of the event amplitude, respectively.
   E) The event is fitted with a 2-level step-fitting algorithm using L_0 and L_1 as levels.
   F) The portion of the event which is best fitted by L_1 is considered as the refined event. If the duration of the event exceeds a user-defined minimum duration, the event is accepted and stored.

3. Event analysis. The refined resistive pulses are analyzed to determine the minimum and the maximum intensity values ($dI_{\mathrm{min}}, dI_{\mathrm{max}}$), which are needed to determine the volume (Λ) and the ellipsoid-equivalent shape (m) of the analyte particles. Since the measurements are affected by noise, the absolute maximum and minimum of the event trace are not good estimates of dI_{min} and dI_{max}. Instead, we developed the following method:

1) A matrix $B_{(R-Q)\times Q}$ is constructed as described above. The 20th and the 80th percentile of each row of B are calculated and stored in two arrays, P_{20} and P_{80}, with sizes $(R-Q)$.

2) The minimum value of the array obtained from the elementwise difference $P_{80} - P_{20}$ is labelled dI_0.
3) The 20th and the 80th percentile of each event, dI_{20} and dI_{80} is computed.
4) $dI_{\min}$ and $dI_{\max}$ are calculated as $dI_{\min} = dI_{20} + dI_0/2$ and $dI_{\max} = dI_{80} - dI_0/2$.
5) $dI_{\min}$ and $dI_{\max}$ are used to compute Λ and m as described in by Yusko *et al.*[6] and Houghtaling *et al.*[7]

Supplementary Note 3: Approximation of protein shape with an ellipsoid of rotation

We developed data analysis software to derive reference values for excluded volume Λ_{r} and length-to-diameter ratio m_{r} of a target analyte form atomic coordinates (*.PDB file):

1) Atomic coordinates of a target protein were extracted from a *.PDB file
2) A grid with lattice dimension *l* was constructed to fully incorporate the protein.
3) The number of cells N_{C} in the grid containing at least one atom of the protein were counted.
4) The volume of the protein was determined as $\Lambda_{\mathrm{r}} = N_{\mathrm{C}} l^3$.
5) All coordinates were rescaled by subtracting their average values ($x_i' = x_i - \bar{x}$, $y_i' = y_i - \bar{y}, z_i' = z_i - \bar{z}$).
6) The equation of an ellipsoid of rotation with volume Λ_{r} was calculated as a function of the axis ratio m_{r}, the coordinates of the center (x_0, y_0 and z_0), and the three Euler angles around the ellipsoid axis (α, β and γ).
7) x_0, y_0, z_0, α, β, γ, and m_{r} were optimized using a non-linear fitting procedure based on a search method to maximize the number of atoms of the protein enclosed within the ellipsoid.

Supplementary Note 4: Impact of ellipsoid shape on the $\Delta I/I_0$ distribution

In the absence of a strong dipole moment and in the case of free rotation inside the nanopore, if a prolate ellipsoid is in the $\Delta I_{\min}/I_0$ orientation, then rotation around two of the three axes will take it away from this orientation, while rotation around the third axis has no effect. In contrast, in the $\Delta I_{\max}/I_0$ orientation, rotation of a prolate around only one of three axes will take it away from this orientation, while rotation around the other two axes has no effect. Since rotation around each axis is equally probable, the $\Delta I_{\min}/I_0$ orientation is less probable than the $\Delta I_{\max}/I_0$ orientation for a prolate particle..

Conversely, for an oblate ellipsoid in the $\Delta I_{\min}/I_0$ orientation, rotation around only one of the three axes will take it away from that extreme orientation. However, in the $\Delta I_{\max}/I_0$ orientation,

rotation around two of the three axes will take it away from this orientation, making this outcome more probable than the other extreme. Therefore, the $\Delta I_{min}/I_0$ orientation is more probable than the $\Delta I_{max}/I_0$ orientation for oblate particles.

Supplementary Figures

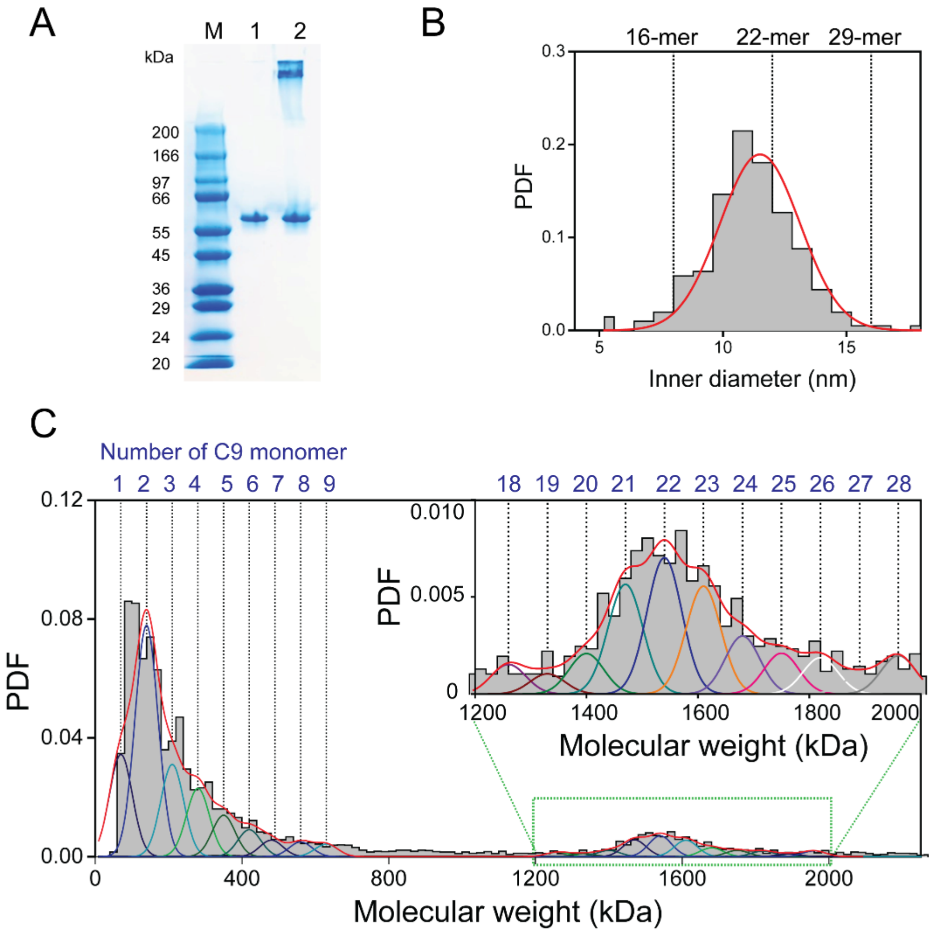

Supplementary Figure S1. Characterization of poly(C9)-amphipol oligomers in solution. A: SDS-PAGE profile of the C9 monomeric solution immediately following buffer exchange into 10 mM HEPES, 50 mM NaCl, pH 7.5. (lane 1) and after overnight incubation with amphipol at 37 °C, resulting in poly(C9)-amphipol oligomers (lane 2) **B:** Histogram of inner pore diameters of poly(C9) rings measured on TEM images (*N* = 205). **C:** Size distribution of C9 by mass photometry. The insert shows zoomed-in events at high mass, indicating 22-mer assembly of C9 (22 x 70 kDa = 1540 kDa) For this calculation, we assumed that the bound amphipol molecules with a molecular weight of ~9 kDa in the poly(C9) amphipol complex has a negligibly small effect on the overall molecular weight of these 1.5 MDa complexes.

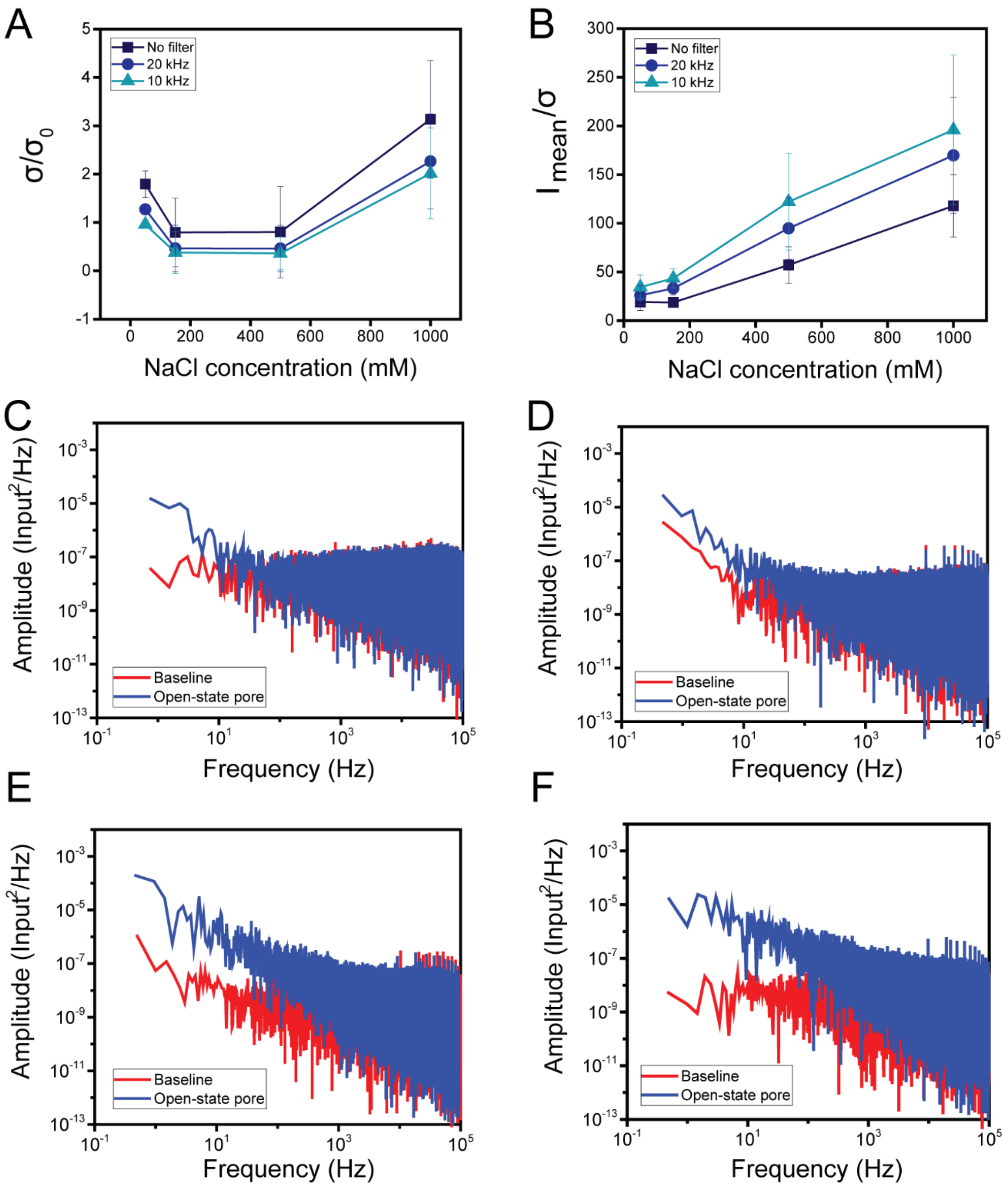

Supplementary Figure S2. Effect of NaCl concentration in the recording buffer on the current noise from recordings with a single poly(C9) pore. A: Relative standard deviation of the recorded current before and after insertion. Here σ is the standard deviation of the open pore current, σ_0 is the standard deviation of the baseline current before pore insertion. **B:** Signal-to-noise ratio; I_{mean} is the mean current of the open pore. Noise characteristics were determined from the current recordings that were collected with a 200 kHz sampling rate with or without Gaussian low-pass filtering. Error bars represent the standard deviation calculated from a minimum of three repeats. Comparison of power spectral densities (PSD) from current before (baseline, red) and after insertion of a single poly(C9) pore (open-state pore, blue) at different NaCl concentrations. **C:** 50 mM, **D:** 150 mM, **E:** 500 mM, and **F:** 1 M with 0.2 µM

amphipol. The PSD was determined from the current recordings that were collected with a 200 kHz sampling rate without filtering.

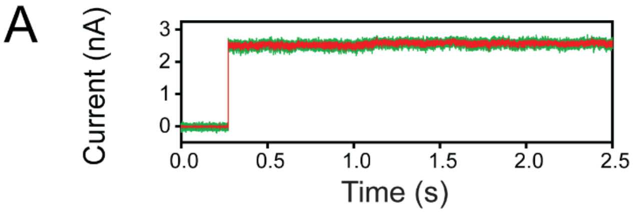

Cutoff frequency	σ/σ_0	I_{mean}/σ
No	7.4±4.8	46.3±11.5
20 kHz	6.2±4.7	59.2±22.7
10 kHz	5.6±4.3	64.5±26.3

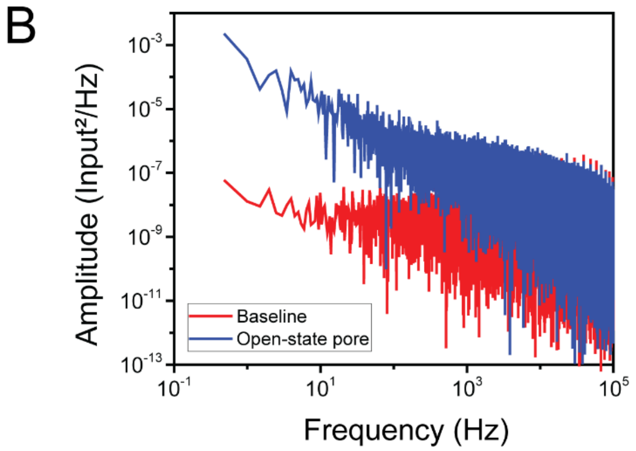

Supplementary Figure S3. Characterization of a single poly(C9) nanopore in the recording buffer with 1 M NaCl without amphipol. A: Single-pore insertion of poly(C9) into lipid bilayers. The current recordings collected with a 200 kHz sampling rate were filtered with a Gaussian low-pass filter with a cutoff frequency of 100 kHz (green) or 10 kHz (red). The relative standard deviation of the signal before and after insertion and the signal-to-noise ratio are shown in the table. Here σ is the standard deviation of the open pore current, σ_0 is the standard deviation of the baseline current before pore insertion and I_{mean} is the mean current of the open pore. **B:** Comparison of power spectral densities (PSD) from current before (baseline, red) and after insertion of a single poly(C9) pore (open-state pore, blue). The PSD was determined from the current recordings that were collected with a 200 kHz sampling rate without filtering.

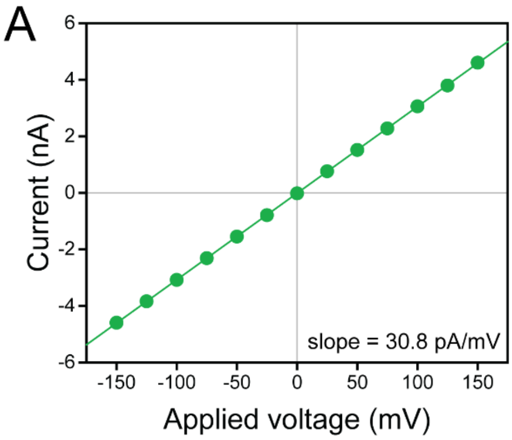

B

Applied voltage (mV)	Current (nA)	SD
150	4.60	0.05013
125	3.80	0.04686
100	3.06	0.06473
75	2.28	0.03463
50	1.52	0.03850
25	0.76	0.03185
0	-0.02	0.02962
-25	-0.79	0.03061
-50	-1.55	0.03274
-75	-2.31	0.03524
-100	-3.08	0.04143
-125	-3.84	0.05414
-150	-4.59	0.05215

Supplementary Figure S4. Current-voltage (I–V) curves of the poly(C9) pore from -150 mV to +150 mV at 1 M NaCl, 0.2 μM amphipol, 10 mM HEPES pH 7.5. **A:** The graph represents an average from three independent repeats. The SD is small, as shown in the table **B**

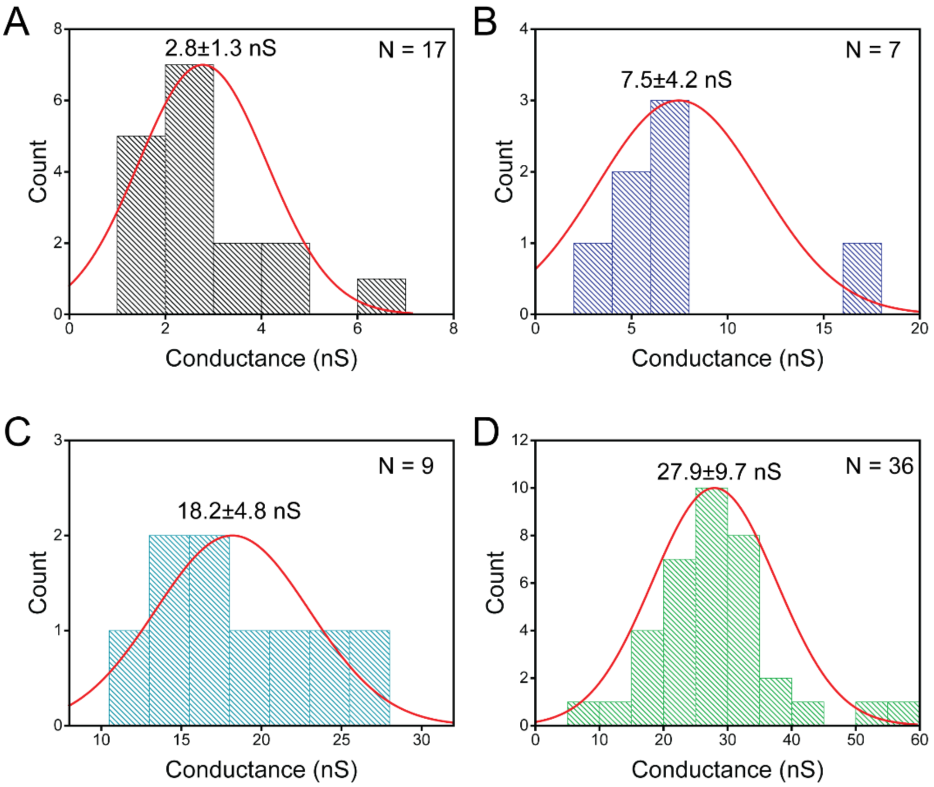

Supplementary Figure S5. Conductance distributions of poly(C9) pores in planar lipid bilayers at four different concentrations of NaCl with a recording buffer containing 10 mM HEPES pH 7.5 and **A:** 50 mM NaCl, **B**: 150 mM NaCl, **C:** 500 mM NaCl, **D:** 1 M NaCl with 0.2 µM amphipol.

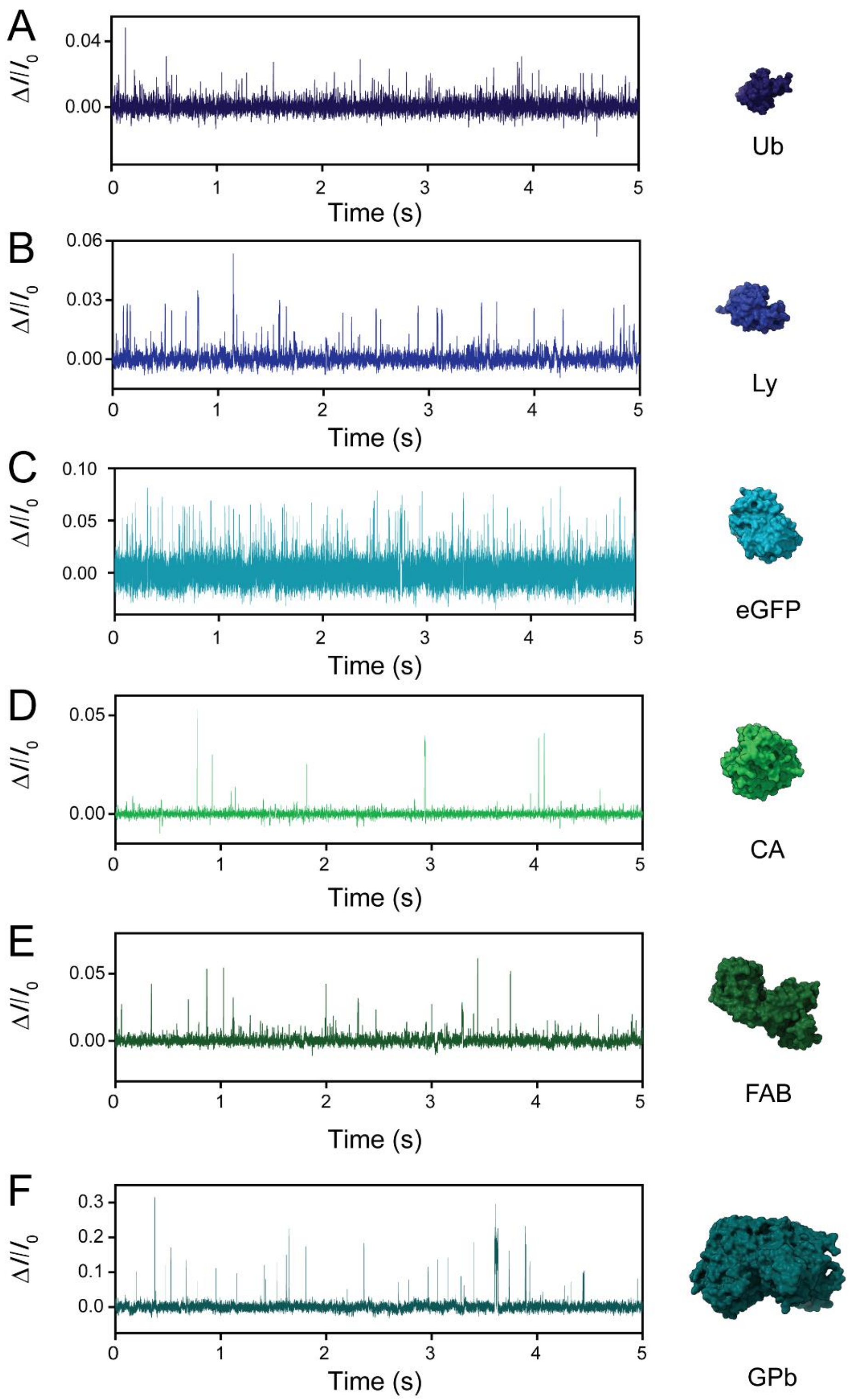

Supplementary Figure S6. Baseline–corrected current recordings showing resistive pulses (upward spikes) measured with poly(C9) nanopores in the presence of **A:** Ub, **B:** Ly, **C:** eGFP, **D:** CA, **E:** FAB, and **F:** GPb. The current recordings were collected in a buffer containing 1 M NaCl, 0.2 µM amphipol, 10 mM HEPES, pH 7.5 with a 200 kHz sampling rate and were filtered with 1 kHz Gaussian low–pass filter for clarity of display.

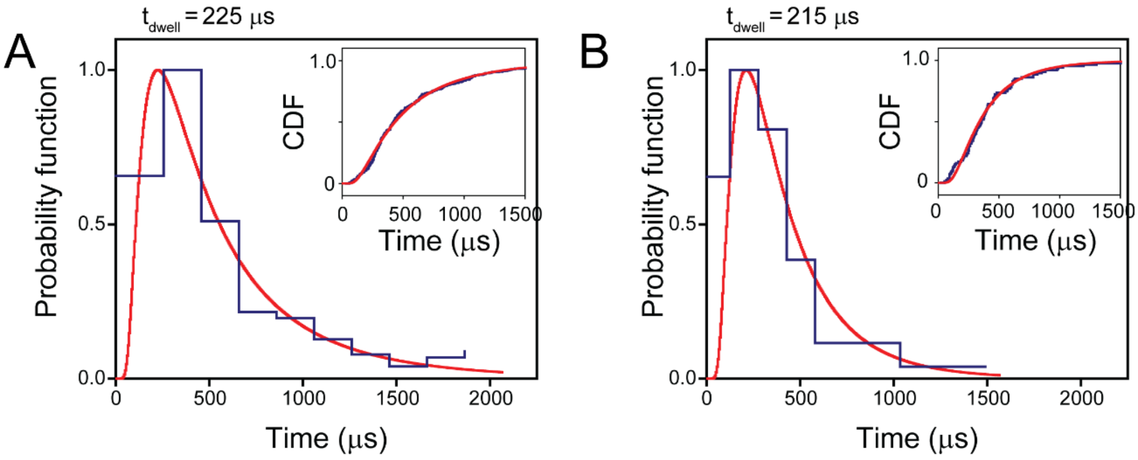

Supplementary Figure S7. The distribution of dwell times t_d of resistive pulses from A: Ly, with the most probable t_d value of 225 µs. The fraction of events exceeding the 450 µs threshold for approximation of the volume and shape of Ly was 0.45. **B:** FAB, with the most probable t_d value of 215 µs. The fraction of events exceeding the 300 µs threshold for approximation of the volume and shape of FAB was 0.60. The distributions include the t_d values of all detected resistive pulses that were longer than 20 µs.

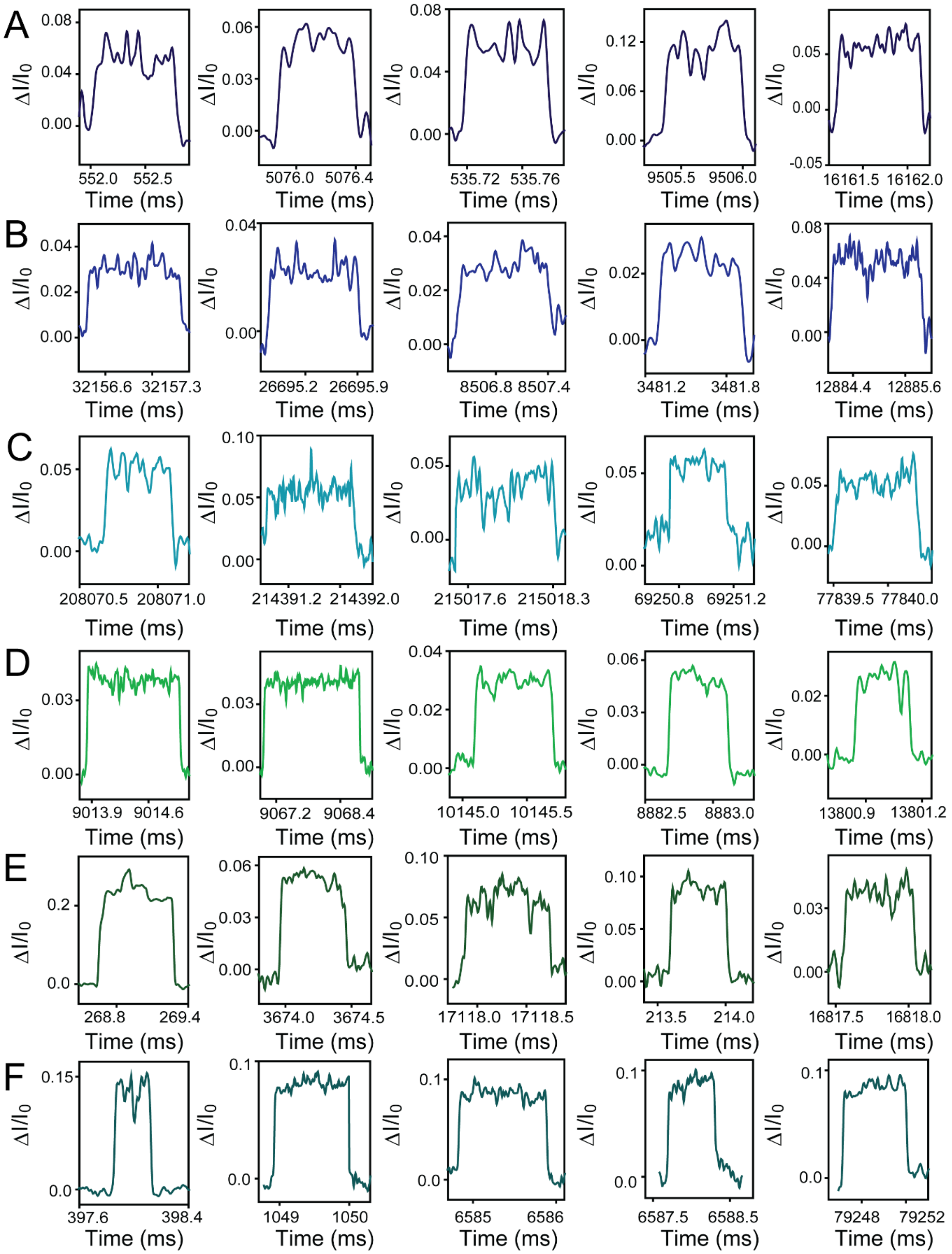

Supplementary Figure S8. Representative individual resistive pulses in the presence of **A:** Ub, **B:** Ly, **C:** eGFP, **D:** CA, **E:** FAB, and **F:** GPb. The current recordings were collected using 1 M NaCl, 0.2 µM amphipol, 10 mM HEPES, pH 7.5 with a 200 kHz sampling rate and were filtered with a 10 kHz Gaussian low-pass filter for Ub and Ly and 20 kHz Gaussian low-pass filter for eGFP, CA, FAB, and GPb.

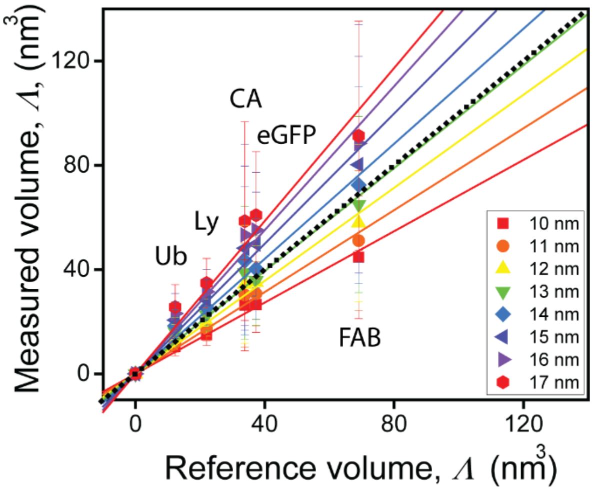

Pore lenght = 13 nm	
slope	Pearson's r
1.056± 0.059	0.992

Supplementary Figure S9. Empirical determination of the effective pore length of poly(C9) pores. Comparison of excluded volume measured in poly(C9) nanopore experiments with the reference volume determined from the atomic coordinates (*.PDB files). We fitted linear regressions with a zero-intercept imposed for accurate analysis. The figure illustrates that an effective pore length of 13 nm most closely matches the ideal slope of 1.0 (black dotted line) of measured values as a function of the reference values of five different proteins.

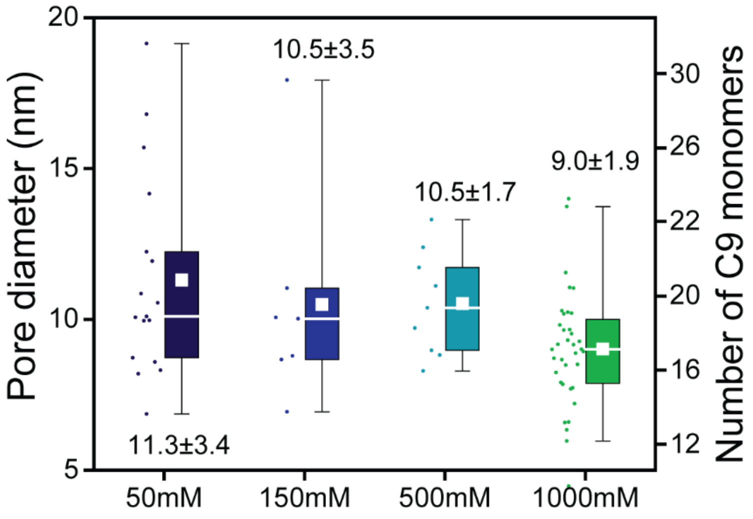

Supplementary Figure S10. Estimations of the effective inner diameter of the poly(C9) pore at different NaCl concentrations. For this calculation, we employed an effective pore length of 13 nm. The mean values are shown by the solid squares. The box range corresponds to the 25th to 75th percentile, whereas the whisker range corresponds to the 5th to 95th percentile.

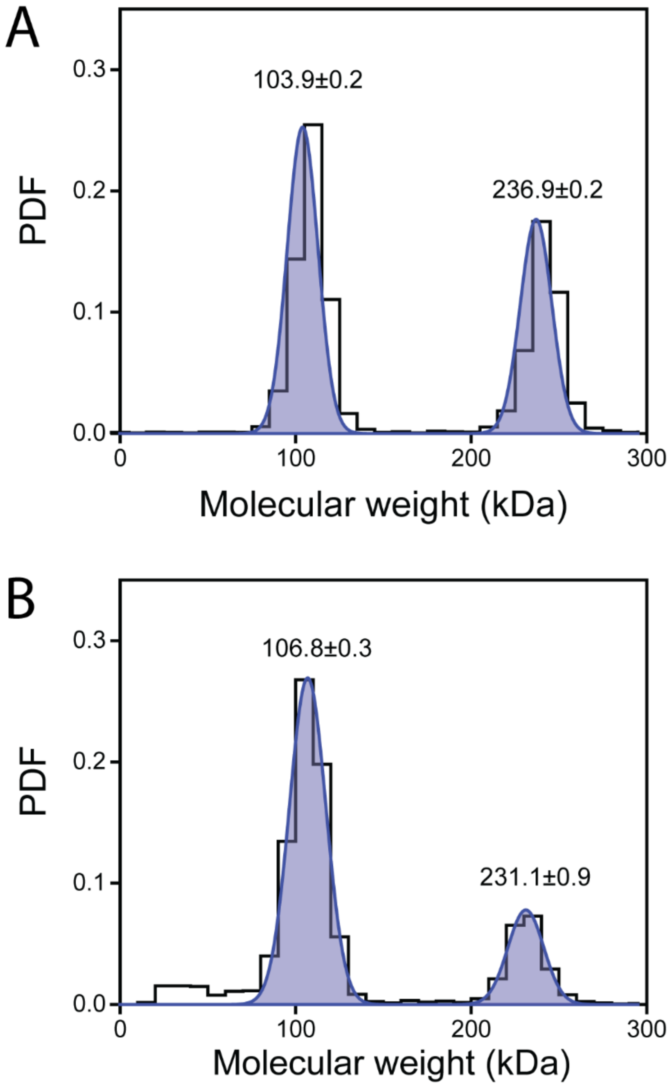

Supplementary Figure S11. Size distribution of a sample containing commercially available GPb by mass photometry recording in two different buffers. A: PBS buffer, pH 7.4, **B:** Nanopore recording buffer containing 1 M NaCl, 0.2 µM amphipol, 10 mM HEPES, pH 7.5. Before this measurement, we calibrated the mass photometry instrument in the respective buffer with molecular weight markers with a range of molecular weights from 20 to 720 kDa.

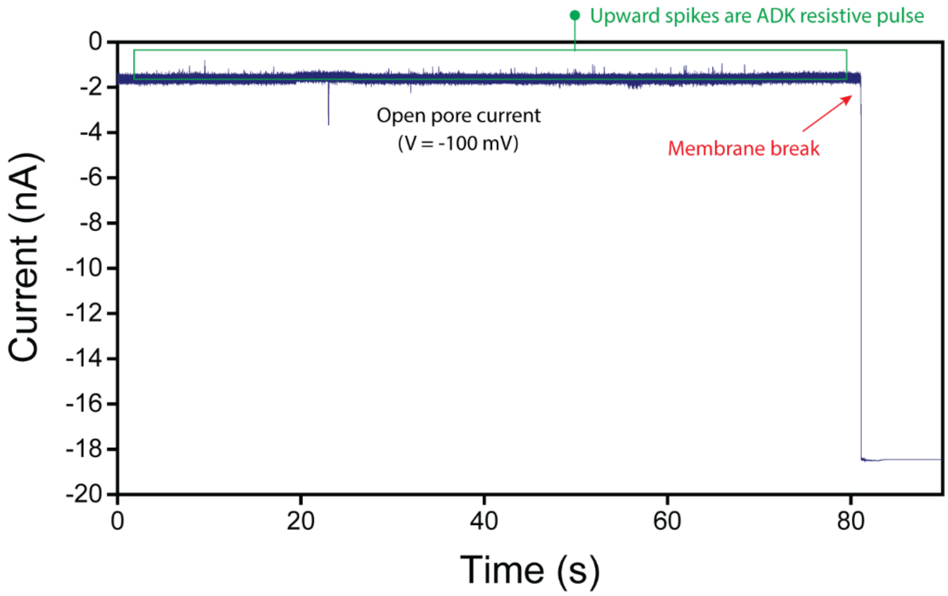

Supplementary Figure S12. Termination of the experiment due to membrane rapture. The trace illustrates spontaneous membrane breaking under an applied potential of –100 mV with a current range of 20 nA.

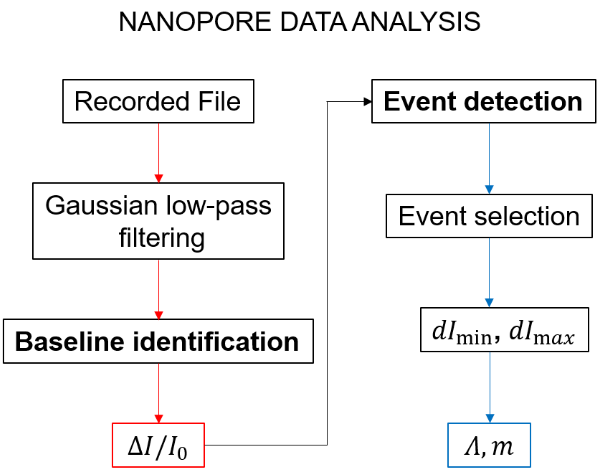

Supplementary Figure S13. Schematic representation of the nanopore data analysis.

Supplementary Tables

Supplementary Table S1. The number, conductance, and diameter of poly(C9) nanopores used in resistive pulse experiments.

Proteins	Number of pores	Conductance (nS)	Pore diameter (nm)
Ub	3	22.1±1.7	7.9±0.4
Ly	3	25.0±3.7	8.5±0.8
CA	3	38.0±20.3	10.8±3.7
eGFP	3	23.0±4.6	8.1±1.0
FAB	3	37.4±9.1	10.9±1.6
GPb	4	35.7±10.3	10.5±1.9
Ub+eGFP+GPb	3	43.6±9.6	12.0±1.7
ADK	5	25.7±8.1	8.6±1.7
ADK+AP5A	3	21.3±7.6	8.1±1.5

Supplementary Table S2. Excluded volumes and length-to-diameter ratios determined by analysis of resistive pulses and their deviations from the reference values.

	**Volume, $\boldsymbol{\Lambda}$**			**Length-to-Diameter Ratio, $\boldsymbol{m}$**		
	Reference volume, Λ_r (nm^3)	Measured volume, Λ (nm^3)	% deviation	Reference m_r	Measured m	% deviation
Ubiquitin	12.3	15.1 ± 3.4	22.8%	1.3	1.5 ±1.0	16.2%
Lysozyme	22	22.8 ± 4.9	3.6%	1.59	1.5 ± 1.0	-3.1%
CA	33.8	38.4 ± 36.8	13.6%	0.8	0.7 ± 0.1	-13.8%
eGFP	37.8	34.5 ± 13.2	-8.7%	1.57	1.6 ± 0.7	2.5%
FAB	69	68.0 ± 46.4	-1.4%	0.6	0.5 ± 0.2	-19.9%
GPb	117.1	116.0±24.8	-0.9%	0.7	0.7±0.2	0
Average deviation of the median			4.8±11.5%	Average deviation of the median		-3.0±12.7

Supplementary Table S3. Excluded volumes and length-to-diameter ratios determined by analysis of resistive pulses of three mixed proteins compared to their reference values.

	**Volume, Λ**		**Length-to-Diameter Ratio, m**	
	Reference volume, Λ_r (nm^3)	Measured volume, Λ (nm^3)	Reference m_r	Measured m
Ub	12.3	11.1 ± 7.6	1.3	1.6 ±1.8
eGFP	37.8	50.7 ± 11.0	1.57	1.9 ± 0.9
GPb	117.1	116.9±24.0	0.7	0.6±0.1

References

(1) Cruickshank, C. C.; Minchin, R. F.; Le Dain, A. C.; Martinac, B. Estimation of the Pore Size of the Large-Conductance Mechanosensitive Ion Channel of Escherichia Coli. *Biophys J* **1997**, *73* (4), 1925–1931. https://doi.org/10.1016/S0006-3495(97)78223-7.

(2) Fennouri, A.; List, J.; Ducrey, J.; Dupasquier, J.; Sukyte, V.; Mayer, S. F.; Vargas, R. D.; Pascual Fernandez, L.; Bertani, F.; Rodriguez Gonzalo, S.; Yang, J.; Mayer, M. Tuning the Diameter, Stability, and Membrane Affinity of Peptide Pores by DNA-Programmed Self-Assembly. *ACS Nano* **2021**, *15* (7), 11263–11275. https://doi.org/10.1021/acsnano.0c10311.

(3) Spicer, B. A.; Dunstone, M. A. Going Full Circle: Determining the Structures of Complement Component 9. *Methods Enzymol* **2021**, *649*, 103–123. https://doi.org/10.1016/bs.mie.2021.01.020.

(4) Spicer, B. A.; Law, R. H. P.; Caradoc-Davies, T. T.; Ekkel, S. M.; Bayly-Jones, C.; Pang, S.-S.; Conroy, P. J.; Ramm, G.; Radjainia, M.; Venugopal, H.; Whisstock, J. C.; Dunstone, M. A. The First Transmembrane Region of Complement Component-9 Acts as a Brake on Its Self-Assembly. *Nat Commun* **2018**, *9* (1), 3266. https://doi.org/10.1038/s41467-018-05717-0.

(5) Dudkina, N. V; Spicer, B. A.; Reboul, C. F.; Conroy, P. J.; Lukoyanova, N.; Elmlund, H.; Law, R. H. P.; Ekkel, S. M.; Kondos, S. C.; Goode, R. J. A.; Ramm, G.; Whisstock, J. C.; Saibil, H. R.; Dunstone, M. A. Structure of the Poly-C9 Component of the Complement Membrane Attack Complex. *Nat Commun* **2016**, *7* (1), 10588. https://doi.org/10.1038/ncomms10588.

(6) Yusko, E. C.; Bruhn, B. R.; Eggenberger, O. M.; Houghtaling, J.; Rollings, R. C.; Walsh, N. C.; Nandivada, S.; Pindrus, M.; Hall, A. R.; Sept, D.; Li, J.; Kalonia, D. S.; Mayer, M. Real-Time Shape Approximation and Fingerprinting of Single Proteins Using a Nanopore. **2017**, *12* (December 2016), 360–368. https://doi.org/10.1038/nnano.2016.267.

(7) Houghtaling, J.; Ying, C.; Eggenberger, O. M.; Fennouri, A.; Nandivada, S.; Acharjee, M.; Li, J.; Hall, A. R.; Mayer, M. Estimation of Shape, Volume, and Dipole Moment of Individual Proteins Freely Transiting a Synthetic Nanopore. **2019**. https://doi.org/10.1021/acsnano.8b09555.
